# Confrontations between *Aspergillus nidulans* and microbial biocontrol agents cause differential regulation of secondary metabolism and synthesis of chemicals toxic to human kidney and colon cells

**DOI:** 10.1128/aem.02180-25

**Published:** 2026-04-13

**Authors:** Bennet Rohan Fernando Devasahayam, Yvonne Poeschl, Henriette Uthe, Robert Rennert, Lena Hartmann, Holger B. Deising

**Affiliations:** 1 Faculty of Nutritional Sciences III, Chair for Phytopathology and Plant Protection, Martin Luther University Halle-Wittenberg, Institute of Agricultural and Nutritional Sciences 9176, Halle, Germany; 2 EcoMetEoR, Molecular Interaction Ecology, German Center for Integrative Biodiversity Research (iDiv), Leipzig, Germany; 3 German Center for Integrative Biodiversity Research (iDiv), Leipzig, Germany; 4 Biometrics and Agricultural Informatics, Faculty of Natural Sciences III, Martin Luther University Halle-Wittenberg 9176, Halle, Germany; 5 Department of Bioorganic Chemistry, Leibniz Institute of Plant Biochemistry, Halle, Germany; The University of Arizona, Tucson, Arizona, USA

**Keywords:** *Aspergillus nidulans*, *Bacillus amyloliquefaciens*, *Bacillus subtilis*, consumer safety, microbial biological control agents (MBCAs), secondary metabolites, toxicity

## Abstract

Microbial interactions in agricultural ecosystems are chemically dynamic, with significant implications for ecological balance and crop protection. As synthetic fungicides face increasing regulatory and resistance challenges, applying microbial biological control agents (MBCAs) presents a potential alternative crop-protection strategy. However, the chemical and toxicological consequences of such applications remain poorly understood. Using the model ascomycete *Aspergillus nidulans*, the response to confrontations with twelve microbial partners, including bacterial and fungal MBCAs, plant pathogens, and phylloplane isolates was studied. Dual-culture assays revealed distinct interaction patterns, and transcriptome profiling showed confrontation-specific activation of secondary metabolite biosynthetic gene clusters (SMBGCs), with up to 50 SMBGCs differentially expressed in *A. nidulans*. Complementary untargeted LC-MS/MS identified hundreds of unique secondary metabolites (SMs), including compounds structurally resembling synthetic fungicides, including azoles and piperidines. Notably, cytotoxicity assays using human HEK-293 and HCT-116 cell lines revealed that SM mixtures from confrontations of *A. nidulans* with *Bacillus subtilis* or *Trichothecium roseum* exhibited significant toxicity, with IC₅₀ values as low as 52 µg/mL. These findings demonstrate that microbial confrontations can trigger the production of diverse and specific sets of SMs, including compounds representing potential human health risks. This study underscores the need for confrontation-informed toxicological assessments in MBCA regulation and highlights the importance of developing safer biocontrol strategies in agriculture.

**IMPORTANCE:**

This study shows fundamental changes at the transcriptome and metabolome level in the ubiquitous fungus *Aspergillus nidulans* confronting various microorganisms, including microbial biological control agents. Strong modulations of transcript abundances of genes belonging to secondary metabolism gene clusters correlated with the formation of a vast array of novel secondary metabolites. Compounds formed in some confrontations were toxic to human cells, questioning the consumer safety of applying microbial biological control agents.

## INTRODUCTION

Current crop protection strategies undergo rigorous transitions, with the establishment of pesticide-free agriculture as one of the prime societal and political goals (1). The European Union has the aim of establishing ecological farming on 25% of the arable area by 2030. Though widely accepted that at least three pesticides addressing distinct targets are needed for each plant–pathogen/pest/weed interaction to avoid or counteract the development of drug resistance, the number of interactions with less than three synthetic chemistries has increased from 510 in the early 1970s to more than 3,500 in 2018 (1, 2). To compensate for these limitations and in agreement with the concept of organic farming, the application of antagonistic microorganisms is envisaged as an appropriate alternative to agrochemicals in crop disease control and to avoid their putative detrimental effects on ecosystems and consumers’ health (3, 4).

In natural environments, an average number of 10^6^–10^7^ bacteria per cm^2^ of leaf surface has been reported, and the complexity of fungal propagules on leaf surfaces, although considerably lower, is still significant (5–7). These multispecies communities establish specific interspecies communication, with secondary metabolites (SMs) playing a key role in this communication (8). Remarkably, a recent analysis of more than 1,000 fungal genomes revealed enormous numbers of secondary metabolite biosynthetic gene clusters (SMBGCs) and significant differences in these numbers across fungi. The class of Eurotiomycetes, including the genera *Aspergillus* and *Penicillium*, has an average of 48 clusters per genome, and, emphasizing variabilities, 25% of the species within this class contain more than 60 clusters. Comparisons of fungal with bacterial genomes and SMBGCs suggested dramatic differences in their biosynthetic logic and space of SMs (8–10). However, the compound complexity based on genome mining and computational predictions may not reflect reality. Most SMBGCs are silent under lab conditions and become activated only under certain environmental conditions, such as in the interaction with other microorganisms. For example, arginine-derived polyketides produced by *Streptomyces* species trigger the production of SMs in cross-kingdom interactions with fungi belonging to the genera *Aspergillus* and *Penicillium* species and may thus, in addition to their direct impact, induce a secondary wave of fungal natural products possibly contributing to the composition of microbial soil communities (11). Moreover, the SM complexity in microbial communities could be further increased via uptake of secreted SMs by other microbes and, depending on their repertoire of SM-modifying enzymes, result in the formation of novel compounds possibly exhibiting increased toxicity (12). These data suggest that the composition of microbial consortia determines both SMBGC activation and formation of various SMs, which, in turn, shape microbial communities (8). Thus, one may hypothesize that microbial biocontrol agents may also dramatically modify the chemical landscape of natural secondary metabolites and thus affect community compositions.

Indeed, this view is supported by field and growth-chamber experiments with soybean seeds coated with the biological control agent *Bacillus cereus*. Analyses of 2,651 individual isolates revealed that bacterial rhizosphere communities from non-treated control plants and from plants grown from seeds coated with a single *B. cereus* strain indicated effects of newly introduced bacteria on community compositions (13). To identify molecular determinants shaping communities, an experimentally tractable tripartite model system consisting of *B. cereus* as a model rhizosphere bacterium and *Pseudomonas koreensis,* as well as *Flavobacterium johnsoniae* co-isolated with *B. cereus,* was employed in pairwise analyses and revealed a hierarchical interstrain-competition network. *P. koreensis*, which is capable of producing a family of alkaloid antibiotics, including koreenceine, inhibited growth of *F. johnsoniae*. In turn, the production of these antibiotics was inhibited by *B. cereus*, and this inhibitory effect is thought to be required for stabilizing the consortium and the formation of more robust biofilms composed of three members as compared to biofilms formed by individual strains (14). Moreover, comparative transcriptomics and metabolomics revealed that the abundance of transcripts of biosynthetic gene clusters differed between mono-, bi-, and tripartite cultures, highlighting that the dynamics of SM formation depend on the community species composition and suggested metabolic handoffs between species (15). The experiments with this defined bacterial model community showed that secondary metabolites are synthesized in a community-determined manner, reinforcing the question of whether or not the introduction of biocontrol bacteria likewise alters the microbial community primarily by the formation of secondary metabolites that may also prove to be toxicologically relevant for consumers.

In a recent study, employing RNA-Seq and non-targeted high-resolution metabolome analyses, we dissected differential transcriptional and metabolomic regulation occurring in bipartite confrontations between a plant pathogenic fungus, i.e., the fungal maize pathogen *Colletotrichum graminicola*, with either the registered biocontrol bacterium *Bacillus amyloliquefaciens* or the ubiquitous fungus *Aspergillus nidulans* (16). When confronting *A. nidulans* with the maize pathogen, 160 SM genes present in 50 SMBGCs were differentially regulated, including 14 polyketide synthase (*PKS*), 14 non-ribosomal peptide synthetase (*NRPS*), 1 *PKS-NRPS* hybrid, and 2 terpene synthase genes. In the PKS cluster responsible for synthesis of the carcinogenic polyketide sterigmatocystin, almost all genes showed increased transcript abundances in RNA-Seq and RT-qPCR analyses. Highlighting the specificity of the responses in the interaction of *Colletotrichum graminicola* with the bacterial or fungal confrontation partner, respectively, only a few confrontation-specific features were commonly detected in both confrontations.

In order to address the plasticity of chemical responses in distance, zone-line, contact, or overgrowth inhibition (17), we established numerous bipartite confrontations between microorganisms naturally occurring in or newly introduced as biocontrol agents into agro-environments. In detail, we investigated confrontations between the ubiquitous fungus *A. nidulans* and three biocontrol bacteria (*Bacillus amyloliquefaciens, Bacillus subtilis, Bacillus velezensis*), three biocontrol fungi (*Trichoderma asperellum, Trichoderma harzianum, Trichothecium roseum*), three plant-pathogenic fungi (*Alternaria tenuis* [syn. *Alternaria alternata*], *Cochliobolus heterostrophus,* and *Colletotrichum higginsianum*), two phylloplane fungi isolated from maize leaves (*Coprinellus domesticus* and a *Cladosporium* species), and a killer toxin-producing strain of the model yeast *Saccharomyces cerevisiae*. RNA-Seq and non-targeted high-resolution LC-MS/MS metabolome studies revealed highly distinct antagonistic responses of *A. nidulans* even to closely related confrontation partners. Pronounced differential regulation of SMBGCs corresponded to the formation of a large number of novel SMs. Importantly, *in vitro* studies employing human cells showed that the mixtures of SMs obtained from confrontations of *A. nidulans* with the microbial biological control agents (MBCAs) *B. subtilis* and *T. roseum* were toxic to healthy human kidney and human colorectal carcinoma cells.

## MATERIALS AND METHODS

### Fungi, bacteria, and plant material used in this study

The ascomycete filamentous fungal strain *Aspergillus nidulans* RMS011 (18) was provided by Dr. Vito Valiante (Hans-Knöll Institute, Jena, Germany). Biocontrol bacteria used in this study were *Bacillus amyloliquefaciens* JKI-BI 7332/2, *Bacillus subtilis* JKI-BI 7325/1, both provided by Dr. Ada Linkies (Julius-Kühn Institute, Institute for Biological Control, D69221 Dossenheim, Germany), and *Bacillus velezensis* NCCB 100737 (Westerdijk Fungal Biodiversity Institute, Utrecht, The Netherlands). Fungal biocontrol strains were *Trichoderma asperellum* JKI-BI 7375 and *Trichoderma harzianum* JKI-BI 7374, both generously provided by Ada Linkies (Julius-Kühn Institute, Institute for Biological Control, Dossenheim, Germany). The K1 killer toxin-producing *Saccharomyces cerevisiae* strain T158C (19) used as a control was kindly provided by Manfred J. Schmitt (Saarland University, Germany). Plant pathogens used as confrontation partners of *A. nidulans* were *Alternaria tenuis* BRFD_At001 (strain collection, Phytopathology and Plant Protection, MLU Halle, Germany); *Cochliobolus heterostrophus* C12 was provided by Stefan G.R. Wirsel (MLU Halle, Halle, Germany); *Colletotrichum higginsianum* Δ*chku70* (20); and *Trichothecium roseum* BRFD1 (strain collection, Phytopathology and Plant Protection lab, MLU Halle, Germany). Two filamentous ascomycetes used in confrontation assays, i.e., *Coprinellus domesticus* BRFD014 and a non-defined species of the genus *Cladosporium* BRFD003, were isolated from leaves of *Zea mays* cv. Mikado is grown in a greenhouse at the Institute of Agricultural and Nutritional Sciences, MLU Halle (Saale), Germany. Leaves of two-week-old maize plants were cut into 1 cm^2^ segments and placed onto potato dextrose agar (PDA; Difco, Fisher Scientific GmbH, Schwerte, Germany) plates (Ø 9 cm). Plates were kept at 25°C for 2 weeks. Genomic DNA of two isolates was prepared using the innuPREP Plant DNA kit (Analytik Jena, Jena, Germany) and Proteinase K-treated as recommended by the manufacturer. ITS sequences were PCR-amplified using primers ITS1 and ITS4 (sequences of these and of other primers used in this study are given in Table S1) (21), and Dream Taq DNA polymerase (Thermo Fisher Scientific, Dreieich, Germany). ITS sequencing, colony, and spore phenotype identified *Cladosporium* spp. BRFD003 and *Coprinellus domesticus* BRFD014.

In addition, both biocontrol bacteria *B. amyloliquefaciens* and *B. subtilis,* and the biocontrol fungus *T. roseum* used for transcriptome studies were subjected to DNA sequencing using the 16S (27F and 1492R) and ITS (ITS1 and ITS4) primers. DNA sequencing was performed by Microsynth Seqlab, and sequence analysis was carried out using BLASTn (https://blast.ncbi.nlm.nih.gov/Blast.cgi) and MEGA11 (22). Phylogenetic analyses were conducted using MrBayes (23). Sequences with high similarity from GenBank were aligned with the query using the MUSCLE algorithm in MEGA11. The optimal evolutionary model was determined using Akaike’s Information Criterion (24), and alignments were converted to NEXUS format for Bayesian inference in MrBayes v3.2.7a. Posterior probabilities were estimated via MCMC sampling, and the resulting phylogenetic tree was visualized using FigTree v1.4.4 (http://tree.bio.ed.ac.uk/software/figtree/).

### Confrontation assays

A mycelial disk (Ø 4 mm) was excised from the margins of 3-week-old PDA plates of *A. nidulans* and inoculated centrally onto fresh PDA plates (Ø 9 cm; Sigma-Aldrich, Darmstadt, Germany). After 7 days at 23°C, the antagonists were inoculated at a distance of 2.5 cm from the margin of the *A. nidulans* mycelium. Solo cultures of all microorganisms were used as controls. Confrontation and control plates were prepared in triplicate and incubated at 23°C for a further 7 days. Plates were photographed, and fungal growth inhibition was calculated by measuring the inhibition distance between two microbial partners at 14 days post-inoculation (dpi). Solo culture(s) of *A. nidulans* and of antagonistic partners were photographed at 14 and 7 dpi, respectively.

### Fluorescence microscopy

Samples for differential interference contrast and fluorescence microscopy were taken from confrontation interfaces of *A. nidulans* at 14 dpi. Samples from solo cultures were taken at 7 dpi. Microscopy was done with a Nikon Eclipse 600 microscope (Nikon, Düsseldorf, Germany). Digital images were taken with a Nikon microscope camera DS-Ri2, and image processing was performed with NIS-Elements imaging software (Nikon, Düsseldorf, Germany). For cell wall-staining, Calcofluor White-M2R (CFW; 1 g/L, Sigma-Aldrich, Darmstadt, Germany) was mixed with 10% (wt/vol) KOH (1:1). For fluorescence microscopy, specimens were incubated with Calcofluor White-M2R for 10 min at room temperature before imaging, and a 40× Plan Fluor lens was used at excitation and emission wavelengths of 350 and 432 nm, respectively, and a laser light transmission of 25% (ND4 in, ND8 in, ND16 in).

### Transcriptomics of *Aspergillus nidulans* under confrontations

For transcriptome profiling, RNA samples of *A. nidulans* confronting *B. amyloliquefaciens*, *B. subtilis,* and *T. roseum* were chosen as fungal-bacterial and fungal-fungal model interactions. Total RNA of *A. nidulans* was isolated using the peqGOLD Total RNA kit (VWR PEQLAB, Darmstadt, Germany), according to the manufacturer’s instructions. The experiment was performed in biological triplicate, and the isolated RNA was purified using the GeneJET RNA Cleanup and Concentration Micro kit (Thermo Scientific, Vilnius, Lithuania). Transcriptome sequencing was performed by Genewiz, Azenta Life Sciences (Leipzig, Germany). High-quality RNA with an RQN of greater than 9 was used for mRNA library construction. Strand-specific RNA-Seq was performed using the Illumina NovaSeq platform (2 × 150 bp paired-end), targeting a depth of 15–20 million paired-end reads per sample for comprehensive transcriptome coverage.

The computational profiling of gene expression from RNA-Seq by identifying the differentially expressed genes (DEGs) was accomplished with the help of the Galaxy Europe server (https://usegalaxy.eu/) (25, 26). Raw-read quality was assessed with FastQC v0.73, and adapter/low-quality reads were trimmed using Trimmomatic v0.38.1. Clean reads were mapped with RNA STAR v2.7.8a to the reference genome of *Aspergillus nidulans* FGSC A4, and based on a gene annotation file (gff), gene counts were generated with FeatureCounts v2.0.1 (NCBI; accessed on 06 February 2024; https://www.ncbi.nlm.nih.gov/datasets/taxonomy/227321/). DEGs were identified using DESeq2 v2.11.40.7, with a cutoff of *P* < 0.05 and an FC > 2 for upregulated, and an FC < 0.5 for downregulated genes. Principal component analyses (PCA) and correlation analyses were also performed with DESeq2, and DEG overlaps were visualized with BioVenn (https://www.biovenn.nl/) (27).

### Mapping of DEGs to secondary metabolite gene clusters

The identified DEGs from all confrontations were mapped to the SMBGCs of *A. nidulans*. A total of 68 SM clusters were drawn using different references (28, 29). AntiSMASH 7.0 (https://fungismash.secondarymetabolites.org/#!/start) (30) was used as an additional independent source to validate the presence of clusters.

### qRT-PCR validation of the *PKS* gene *stcA*

The expression of the PKS gene *stcA* (accession number ANIA_07825) was studied by qRT-PCR. Primers were designed based on the *stcA* sequence from NCBI, using Serial Cloner 2-6-1 (FileHippo.com) and Clone Manager 9 (https://scied.com/in_conta.htm) software. Actin was used as the housekeeping gene. The experiment was carried out using the iTaq Universal SYBR Green One-Step kit (Bio-Rad Laboratories, California, USA), with three independent biological and three technical replicates. The melting curve was used to validate primer specificity. The relative-fold change in transcript abundance was calculated using the 2^−ΔΔCt^ method (31).

### Metabolite extraction

Secondary metabolites were extracted from PDA plates as described (32), with some modifications. At 14 dpi, 5 mm wide and 5 mm long PDA stripes from the margins of solo cultures or confronting colonies were excised from 100 petri dishes each and transferred to an Erlenmeyer flask. In addition, PDA stripes from control zones without microbial cells were excised. Compounds were extracted as described (33), and extracts were dried in a rotary evaporator (Laborota 4000, Heidolph Instruments GmbH, Schwabach, Germany) at 45°C and 240 mbar. The resulting dry crude extracts were adjusted to a concentration of 1 mg/mL in methanol.

### LC-MS/MS analysis

Liquid chromatography electrospray ionization quadrupole time-of-flight mass spectrometry (LC-ESI-Q-ToF-MS) was performed as described (34). Chromatographic separation was carried out at 40°C using an UltiMate 3000 RSLC system (Thermo Fisher Scientific, Vilnius, Lithuania) fitted with an Acclaim RSLC 120 column (150 × 2.1 mm, 2.2 µm particle size). The gradient employed a 0.4 mL/min flow rate: 0–1 min, 95% solvent A (water/formic acid 99.95/0.05 [vol/vol]) and 5% solvent B (acetonitrile/formic acid 99.95/0.05 [vol/vol]); 1–2 min, linear shift to 20% B; 3–8 min, to 25% B; 8–16 min, to 95% B; 16–18 min, isocratic 95% B; 18–18.01 min, back to 5% B; and held until 20 min. The injection volume was 3 µL (full-loop mode). Mass spectrometric detection was performed on a maXis impact UHR-Q-ToF-MS (Bruker Daltonics, Bremen, Germany) with Apollo II ESI source in positive mode, scanning m/z 90–1,600 at 5 Hz. Calibration was done individually using sodium formate cluster ions infused post-run (1.66 µL/min of 10 mM sodium formate in isopropanol/water with 0.2% wt/vol formic acid).

Raw LC-MS and MS/MS data were processed using MetaboScape 4.0 (Bruker Daltonics), applying the T-ReX 3D algorithm with intensity threshold = 1,500 counts; minimum peak length = 7 spectra; signal = intensity; mass recalibration = auto. Recursive feature extraction settings included a minimum recursive peak length of 5 spectra and presence in at least 1 of 8 analyses.

Feature annotation employed (i) an in-house library with known retention time, mass, and spectra; (ii) KNApSAcK database (35); and (iii) spectral matching with public databases (NIST17, WEIZMASS, Sumner Library, MoNA, GNPS, ReSpect) and Bruker’s internal library.

Compound class prediction was achieved via SIRIUS (v5.8.6), integrating CSI:FingerID, CANOPUS, and ZODIAC. *De novo* molecular formula prediction used: adducts [M+H]^+^, [M+Na]^+^, [M+K]^+^; isotope filtering; 5 ppm MS2 tolerance; up to 10 candidates per spectrum; 60 s tree timeout; with heuristics above 300 m/z. ZODIAC scoring was set to evaluate 10 (300 m/z) to 50 (800 m/z) candidates, using a 2-step Gibbs sampling method (20,000 iterations, 2,000 burn-in, 10 runs). CSI:FingerID used fallback adducts and searched across databases, including KEGG, PubChem, CHEBI, GNPS, and COCONUT.

### Post-processing of LC-MS data

The resulting feature table was post-processed in R (36) by removing features found in blanks (ACN) and growth medium (PDA). Intensities below 1,500 were set to zero, matching the MetaboScape intensity threshold. Features overlapping with solo cultures of at least one of the confrontation partners were also excluded to isolate confrontation-specific features. A resulting feature table was used for comparative analysis, with violin and UpSet plots used for visualization (37) (http://doi.org/10.5281/zenodo.3700590).

Each unique pair of m/z and rt was treated as a distinct feature or putative compound. Annotated features were classified by chemical class and considered (putative) compounds when supported by spectral database matches.

### Assessment of cytotoxicity of confrontation-derived metabolites on human cell lines

The human cell lines HEK293, healthy embryonic kidney cells, and HCT-116, colorectal cancer cells, were used in this study for *in vitro* cell viability assays, proving the impact of metabolic extracts from confrontation cultures on the viability and proliferation of human cells. The HEK293 and HCT-116 cell lines were purchased from ATCC (Manassas, VA, USA) and DSMZ (Braunschweig, Germany), respectively.

MEM (high glucose) and McCoy’s 5A basal media, fetal calf serum (FCS), L-glutamine (200 mM), phosphate-buffered saline (PBS), and 0.05% (wt/vol) trypsin-EDTA were purchased from Capricorn Scientific GmbH (Ebsdorfergrund, Germany). Trypan blue was purchased from Invitrogen (Waltham, MA, USA). Resazurin and digitonin were from Sigma Aldrich (Taufkirchen, Germany), and DMSO from Duchefa Biochemie (Haarlem, The Netherlands). Cell culture and other lab plastics were purchased from TPP (Trasadingen, Switzerland), Greiner Bio-One (Frickenhausen, Germany), and Sarstedt (Nümbrecht, Germany).

The cells were cultivated in their specific growth medium, i.e., basal medium supplemented with 10% (vol/vol) heat-inactivated FCS and 2 mM L-glutamine. The basal medium for HEK293 was MEM (high glucose), while for HCT-116, McCoy’s 5A was used. Cultivation was routinely done in T-75 flasks in a humidified atmosphere with 5% (vol/vol) CO_2_ at 37°C to reach subconfluency (~70%–80%) prior to subsequent sub-culturing or assay usage, when the adherent cells were rinsed with PBS and detached by using trypsin/EDTA (0.05% wt/vol in PBS) prior to cell passaging and seeding.

The impact of the extracts under investigation on the viability and proliferation of both human cell lines was investigated by conducting fluorometric resazurin-based cell-viability assays, using a protocol established in our lab (38, 39). In brief, the human cells were seeded at low densities in 96-well plates—i.e., 6,000 cells/well with HEK293 and 10,000 cells/well with HCT-116—yielding a seeding confluency of ~10%–20%, and were allowed to adhere overnight. Subsequently, the cells were treated for 48 h with the metabolic extracts of *A. nidulans* confronted by *B. amyloliquefaciens*, *B. subtilis*, and *T. roseum*. Extracts of non-confronted *A. nidulans* and of non-colonized PDA served as a control. Extracts were used in a dose range of 0.98–250 µg/mL. For control measures, cells were treated with 0.5% (vol/vol) DMSO (negative control, representing the final DMSO content of the highest concentrated extract samples, for data normalization set to 100% cell viability) and 100 µM digitonin (positive control, for data normalization set to 0% cell viability). After 48 h of incubation, the incubation media were discarded, and cells were rinsed once with PBS. Resazurin was prepared in basal medium and added to the cells at a concentration of 50 µM. Subsequently, the cells were incubated under standard growth conditions for a further 2 h. Finally, the conversion of resazurin to resorufin by the remaining viable cells was fluorometrically measured (λexc. 540 nm/λem. 590 nm) by using a SpectraMax iD5 multi-well plate reader (Molecular Devices, San Jose, USA). Data were determined in at least four biological replicates, each with technical triplicates. IC_50_ curves and values were calculated by using a four-parametric function and GraphPad Prism v10.1 software (San Diego, CA, USA).

### Statistical analyses

If not otherwise noted, data represent means from three independent biological replicates. Statistical differences between groups were analyzed using a single-factor ANOVA test, followed by a Tukey-HSD test, with an alpha degree of *P* < 0.05. Data were assessed with Excel 2016 and R (36) (https://www.R-project.org/), along with the package ggplot2 (40) for generating violin plots and bar plots.

## RESULTS

### The ubiquitous model ascomycete *Aspergillus nidulans* shows distinct interaction patterns with bacteria and fungi, exhibiting distinct lifestyles

Microbes exhibit neutralistic, mutualistic, commensalistic, pathogenic, or antagonistic interactions. In confrontation assays on agar plates, antagonistic interactions have been categorized as distance, zone-line, contact, and overgrowth inhibition (41, 42). Confrontations exhibiting distance or zone-line inhibition are likely due to the production and secretion of antimicrobial molecules, with the size of the inhibition zone primarily depending on the toxicity and diffusion constant of the inhibitory compound(s). We studied the interactions of *A. nidulans* RMS011 (18) with three biocontrol *Bacillus* species, i.e., *B. amyloliquefaciens* JKI-BI 7332/2, *B. subtilis* JKI-BI 7325/1 (Fig. S1A, D, and G, and S1B, E, and H), and *B. velezensis* NCCB 100737 (43), with three biocontrol fungi, i.e., *Trichoderma asperellum* JKI-BI 7375, *Trichoderma harzianum* JKI-BI 7374, and *Trichothecium roseum* BRFD1 (Fig. S1C, F, and I) (44), and with the plant-pathogenic fungi *Alternaria tenuis* BRFD_At001 (syn. *A. alternata*) (45), *Cochliobolus heterostrophus* C12 (46), and *Colletotrichum higginsianum* Δ*chku70* (47). To test the interaction with species naturally occurring on leaf surfaces, we used two fungi isolated from a leaf of *Zea mays* cv. Mikado. ITS fragments of approx 600 and 500 bp were obtained by PCR amplification, and sequences of these fragments identified one isolate as *Coprinellus domesticus* BRFD014, based on BLASTn comparisons (Fig. S2B and D). The ITS sequence of the other isolate showed 90% identity to those of *Cladosporium allicinum* and *Cladosporium floccosum*. As we were not able to unequivocally assign the ITS sequence to either of these fungi, the second isolate will be referred to as *Cladosporium* spp. BRFD003 (Fig. S2A and C) in this study. The K1 killer toxin-producing *Saccharomyces cerevisiae* strain T158C (19) caused toxin-mediated distance inhibition and served as a control.

**Fig 1:**
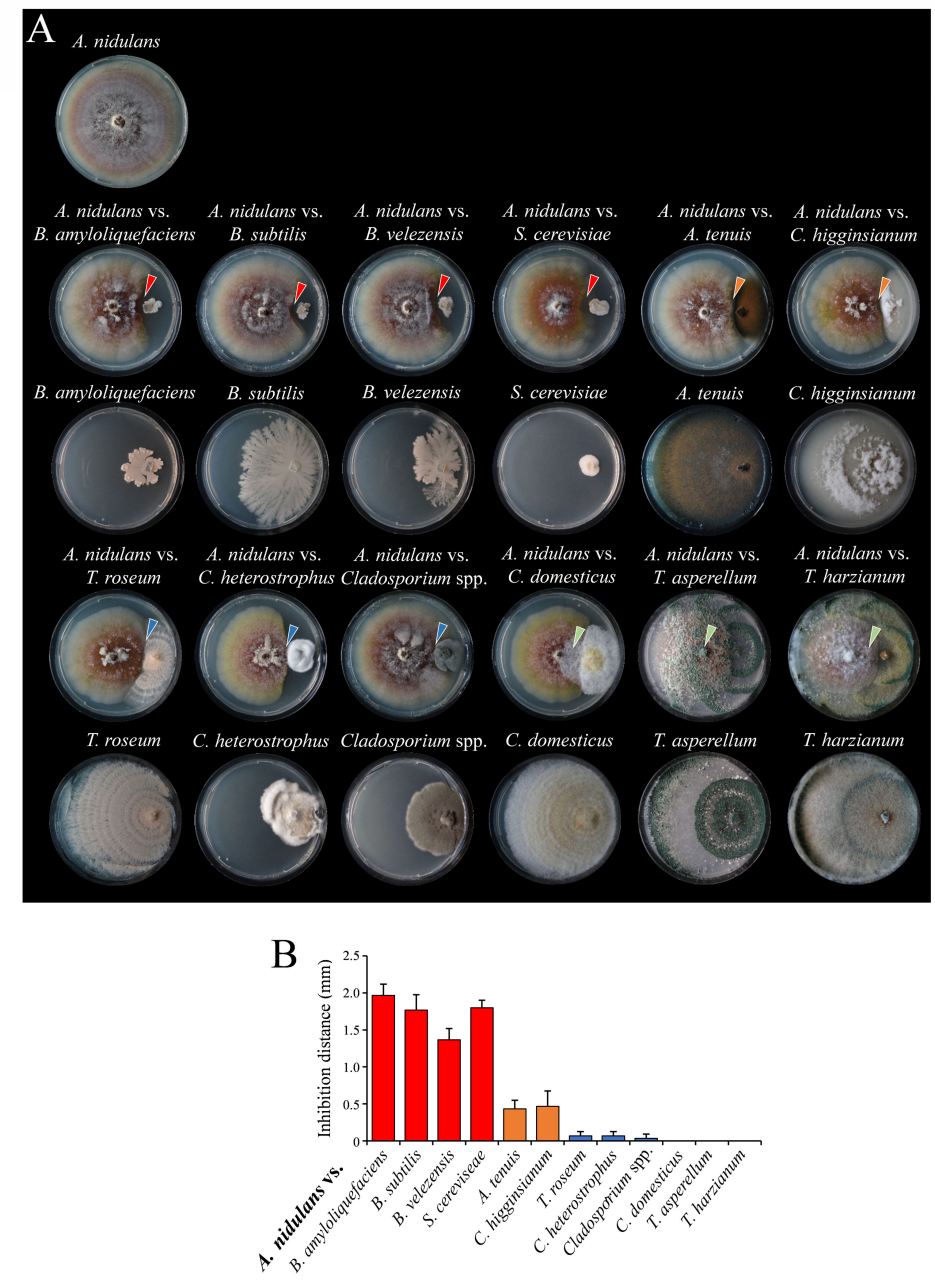
Confrontation assays of *Aspergillus nidulans* with various bacteria and fungi reveal diverse interaction patterns. (**A**) Representative confrontation assays showing interactions between *A. nidulans* and a range of bacterial and fungal biocontrol strains, including plant pathogens. Clear inhibition zones (red arrowheads) were observed in interactions with *Bacillus amyloliquefaciens*, *B. subtilis*, *B. velezensis*, and *Saccharomyces cerevisiae*, suggesting distance-based antagonism. Zone-line inhibition (orange arrowheads) was observed in confrontations between *A. nidulans* with *A. tenuis* and *C. higginsianum*. Contact-dependent inhibition (blue arrowheads) was evident in the confrontation with *Trichothecium roseum*, *Cochliobolus heterostrophus,* and *Cladosporium* spp., where colony growth was halted upon hyphal contact. In contrast, overgrowth (green arrowheads) was observed in interactions with *Coprinellus domesticus*, *Trichoderma asperellum*, and *T. harzianum*, where the confrontation partner overgrew the *A. nidulans* colony. For each interaction, solo cultures of *A. nidulans* (top left) and the respective partner species (directly below each confrontation plate) are shown. All images were captured at 14 dpi. Images were captured at 14 dpi for *A. nidulans* solo and confrontation cultures, and at 7 dpi for antagonistic control plates. (**B**) Quantification of inhibition distances measured between *A. nidulans* and the respective confrontation partners at 14 dpi. Red bars denote interactions exhibiting distance inhibition, orange bars indicate zone-line inhibition, and blue bars represent contact inhibition. Data represent means from three independent biological replicates, and error bars indicate standard deviation (+SD).

All confrontations between *A. nidulans* and the established biocontrol agents *Bacillus* species, i.e., *B. amyloliquefaciens*, *B. velezensis*, and *B. subtilis*, showed clear inhibition zones of up to ~2 mm between the colonies, indicative of distance inhibition (Fig. 1A and B*, A*. *nidulans* vs *B. amyloliquefaciens*, *B. subtilis*, and *B. velezensis*, red arrowheads). Importantly, not only the growth of the *A. nidulans* mycelium but also of the *Bacillus* strains was strongly restricted (Fig. 1A). Interestingly, the inhibition zone between *A. nidulans* and the killer toxin-producing yeast *S. cerevisiae* was comparable to the zone between *A. nidulans* and the *Bacillus* strains (Fig. 1A and B*, A*. *nidulans* vs *S. cerevisiae*, red arrowhead). Also, the confrontations between *A. nidulans* and the plant pathogens *A. tenuis* and *C. higginsianum* resulted in distance inhibition, though the size of the inhibition zones was smaller than in the confrontation with the biocontrol bacteria. Hence, one might alternatively categorize this inhibition type as zone-line inhibition (Fig. 1A*, A*. *nidulans* vs *A. tenuis* and *C. higginsianum*, arrowheads; Fig. 1B). Distance inhibition and growth restriction of both partners suggest secretion of antimicrobial compounds by *A. nidulans*, as well as by the confrontation partners. Indeed, the *Bacillus* species used in this study produce non-ribosomal cyclic lipopeptides such as the iturins, which cause fungal cell wall defects as indicated by large swellings (48), and bright-field and fluorescence microscopy of Calcofluor-stained hyphae of *A. nidulans* confronting *B. amyloliquefaciens*, *B. velezensis*, and *B. subtilis* revealed protrusions (Fig. S3A, *A. nidulans* vs *B. amyloliquefaciens*, *B. velezensis*, or *B. subtilis*, arrowheads; Fig. S3B and C). The interactions between *A. nidulans* and *T. roseum*, *C. heterostrophus*, and the *Cladosporium* spp. isolated from maize resulted in contact inhibition, characterized by the arrest of growth upon direct contact between the microbial partners (Fig. 1A and B*, A*. *nidulans* vs *T. roseum*, *C. heterostrophus*, and *Cladosporium* spp.). Also, in confrontations with zone-line and contact inhibition, i.e., in confrontations with fungi such as *A. tenuis*, *T. roseum,* and the *Cladosporium* isolate, hyphae of *A. nidulans* exhibited protrusions, but these were clearly distinct from those caused by *Bacillus* species. In these fungus-fungus confrontations, protrusions were smaller and often appeared as lateral protuberances (Fig. S3A; *A*. *nidulans* vs *A. tenuis*, *T. roseum*, *Cladosporium* spp.; arrowheads; Fig. S3B and C). Interestingly, also hyphae of *A. nidulans* confronting the yeast *S. cerevisiae* showed some weak hyphal swellings possibly due to the viral killer toxin K1 of yeast strain T158C (19, 49) (Fig. S3A; *A. nidulans* vs *S. cerevisiae*, arrowheads). Other confrontation partners, such as the pathogens *C. heterostrophus*, the basidiomycete *C. domesticus,* and the biocontrol fungus *T. asperellum* (50), caused no swellings but hyphal stunting and hyperbranching in *A. nidulans* (Fig. S3A, arrows; Fig. S3B and D). Furthermore, hyphal protrusions or hyperbranching were observed in specific confrontations and never occurred together (Fig. S3B).

In the interactions between *A. nidulans* and *C. domesticus*, growth of *C. domesticus* was restricted upon immediate contact with the *A. nidulans* colony, but growth inhibition was incomplete and *C. domesticus* partially overgrew the *A. nidulans* mycelium (Fig. 1, *A. nidulans* vs *C. domesticus*, green arrowhead). Both *Trichoderma* species overgrew the *Aspergillus* colonies almost completely, formed aerial mycelium abundantly, and represented typical examples of overgrowth-type inhibition (Fig. 1A, *A*. *nidulans* vs *T. asperellum; A. nidulans* vs *T. harzianum*, green arrowheads). Hyphae of solo cultures of *A. nidulans* and of all bacterial and fungal strains used in confrontations did not show any morphological abnormalities (Fig. S4).

Phenotypic changes observed during microbial interactions may be associated with differential regulation of SMBGCs and transcriptomic and metabolomic remodeling in response to interspecies chemical signaling (8, 51). Hence, we decided to analyze the transcriptome of *A. nidulans* growing in confrontation with the MBCAs *B. amyloliquefaciens*, *B. subtilis*, and *T. roseum*.

### *A. nidulans* shows a specific transcriptional response to distinct confrontation partners

In order to investigate the specificity of the transcriptional response of *A. nidulans* to distinct confrontation partners, mRNA was isolated from the 5 mm mycelial edge of *A. nidulans* confronting two approved bacterial biocontrol species, i.e., *B. amyloliquefaciens* and *B. subtilis*, both of which exhibited distance inhibition (Fig. 1) and both of which cause severe swellings in hyphae of *A. nidulans* (Fig. S3A through C). In addition, transcripts were isolated from the 5 mm mycelial edge of *A. nidulans* contacting *T. roseum* (Fig. 1), a fungus with dual, i.e., pathogenic and biocontrol lifestyles (52). Samples taken from 5 mm of non-confronting mycelial borders of solo cultures of *A. nidulans* served as controls. The three independent biological replicates of the *A. nidulans* solo culture, of cultures confronting the two biocontrol bacteria and the culture confronting *T. roseum,* yielded 12 libraries, the sequencing of which generated a total of 384.36 million raw reads, and 373.6 million clean reads were obtained after trimming and quality control. A total of 97.08% of the clean reads were successfully mapped to the reference genome of *A. nidulans* FGSC A4 (NCBI; accessed on 14 September 2022; https://www.ncbi.nlm.nih.gov/genome/17?genome_assembly_id=299190) and corresponded to a total of 10,771 genes. DEGs were identified, based on a cutoff value of *P* < 0.05 and increased (FC > 2) or reduced (FC < 0.5) transcript abundances. The detailed data of transcriptome profiling are provided in Table S2.

PCAs revealed that the samples of the solo culture and of each distinct confrontation clustered closely, indicating uniformity and dependability of the independent replicates of each sample group (Fig. S5A). The distinctiveness across the groups is further highlighted by a heatmap of the correlation matrices, with sample clustering based on normalized transcript counts (Fig. S5B). A total of 591, 1,126, and 1,840 DEGs were obtained for *A. nidulans* confronting *B. amyloliquefaciens*, *B. subtilis,* and *T. roseum*, respectively. Surprisingly, *B. subtilis* caused differential regulation of almost twice as many genes in *A. nidulans* as the closely related bacterium *B. amyloliquefaciens*. Of the 591 and 1,126 DEGs of *A. nidulans* confronting *B. amyloliquefaciens* or *B. subtilis*, respectively, 439 and 799 genes showed increased transcript abundances, and 152 and 327 genes exhibited reduced transcript abundances, respectively. In the interaction between *A. nidulans* and *T. roseum*, an even larger number of DEGs was identified, with 978 genes showing increased and 862 decreased transcript abundances (Fig. S5C). The detailed summary for all DEGs in all confrontations is provided in Tables S3 to S5. Intriguingly, the Venn diagram (Fig. S5D) revealed 266, 353, and 1,037 unique DEGs in the interactions of *A. nidulans* with *B. amyloliquefaciens*, *B. subtilis*, and *T. roseum*, respectively, and a total of only 91 genes commonly differentially regulated in all confrontations were identified (Table S6). Collectively, these data strongly suggest that *A. nidulans* discriminates between, and responds distinctively to, individual bacterial and fungal challenges.

**Fig 2:**
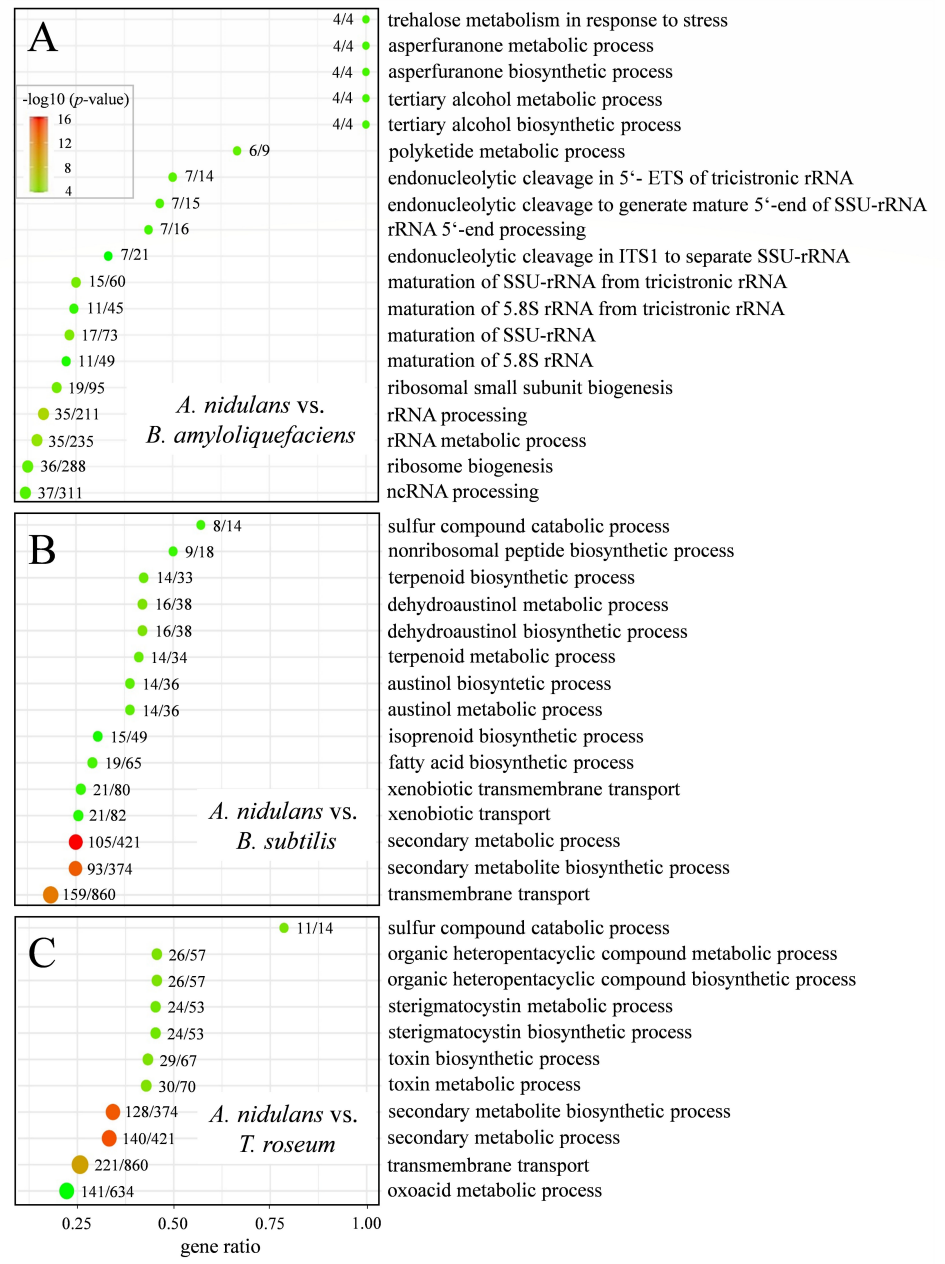
GO enrichment analysis of DEGs in *A. nidulans* under microbial confrontations. Bubble plots representing enriched GO biological process (GO:BP) terms among DEGs in *A. nidulans* during confrontations with (**A**) *Bacillus amyloliquefaciens*, (**B**) *B. subtilis*, and (**C**) *Trichothecium roseum*. The X-axis shows the gene ratio, calculated as the number of DEGs annotated to a given GO term relative to the total number of *A. nidulans* genes associated with that term. Bubble size corresponds to the number of DEGs involved in each GO category. Color gradient reflects the level of statistical significance, indicated as the −log_10_ of the adjusted *P* value. Only GO terms with adjusted *P* < 0.05 were included.

In order to attribute differential gene expression to functional categories in distinct confrontations, functional enrichment of DEGs was carried out using Gene Ontology (GO; geneontology.org) analyses with the classification term biological processes (GO:BP). In confrontation with the biocontrol bacterium *B. amyloliquefaciens*, 270 differentially regulated genes involved in 19 biological processes were identified in *A. nidulans* (Fig. 2A; Table S7, *A. nidulans* vs *B. amyloliquefaciens*). Remarkably, multiple GO terms related to ribosomal RNA processing, ribosome biogenesis, and rRNA metabolic processes were significantly enriched, highlighting a major shift in translational activity in *A. nidulans*. While the precise number of genes involved across these processes may vary by category, the collective enrichment suggests substantial translational activity in *A. nidulans* in response to microbial interaction. In addition to ribosome biogenesis in a broad sense, secondary metabolism is prominently altered at the transcriptional level, as indicated by asperfuranone biosynthesis and metabolism (53), as well as polyketide metabolism (Fig. 2A; Table S7). In further support of an alteration in secondary metabolism, tertiary alcohol biosynthesis and metabolism were also differentially regulated (Fig. 2A). Tertiary alcohols play a key role in metabolism of sesquiterpenoid toxins, as, for example, synthesized in the plant pathogen *Botrytis cinerea* (54). However, the high gene ratio observed is based on a small reference gene set (e.g., only four genes in total), all of which are present in our RNA-seq data set. While this yields a 100% gene ratio, such values should be interpreted with caution, as they may not necessarily indicate strong biological enrichment but rather reflect the small size of the annotated gene category.

By contrast, in the confrontation between *A. nidulans* and *B. subtilis,* the GO:BP terms indicated massive transcriptional differential regulation of secondary metabolism, but neither rRNA formation and processing nor ribosome biogenesis was significantly affected, highlighting striking differences between the interactions of *A. nidulans* with the two closely related bacteria (Fig. 2A and B). Instead, in the *A. nidulans* vs *B. subtilis* confrontation, GO:BP terms suggested strongly differentially regulated non-ribosomal peptide biosynthesis, isoprenoid and, plausibly, terpenoid biosynthesis and metabolism, as well as austinol/dehydroaustinol biosynthetic and metabolic processes. Altogether, a total of 538 differentially regulated genes involved in 15 different biological processes were identified in the confrontation between *A. nidulans* and *B. subtilis* (Fig. 2B; Table S7, *A. nidulans* vs *B. subtilis*). Moreover, in hyphae of *A. nidulans* confronting the fungus *T. roseum*, secondary metabolism was found to be highly differentially regulated, as reflected by the enrichment of GO:BP categories. The most prominently affected processes include the biosynthesis and metabolism of organic heteropentacyclic compounds, toxins, and secondary metabolites, and, most importantly, sterigmatocystin biosynthesis and metabolism. Sterigmatocystin is a polyketide closely related to aflatoxins and is, like aflatoxins, considered a carcinogen (55, 56). In total, 800 differentially regulated genes associated with 11 biological processes were identified in this interaction (Fig. 2C; Table S7, *A*. *nidulans* vs *T. roseum*). In the confrontations with the biocontrol bacterium *B. subtilis* and the biocontrol fungus *T. roseum*, the functional enrichment of transcripts associated with transmembrane transport suggests increased membrane transport activity, potentially including the export of secondary metabolites (Fig. 2B and C; Table S7 *A*. *nidulans* vs *B. subtilis* and *A. nidulans* vs *T. roseum*).

Collectively, transcriptomic and functional enrichment analyses revealed pronounced differences in the metabolic responses of *A. nidulans* to distinct biocontrol partners. These findings highlight the partner-specific modulation of its secondary metabolism during microbial confrontations.

### Distinct confrontation partners cause distinct patterns of differential regulation of SMBGCs in *A. nidulans*

**Fig 3:**
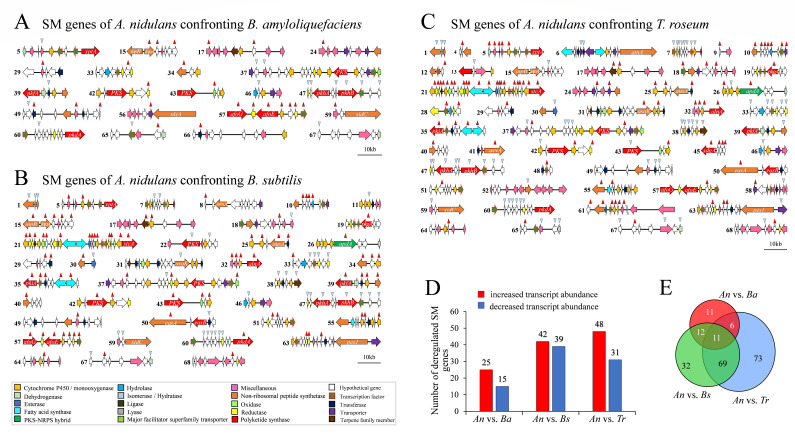
Global transcriptional changes of SMBGCs in *A. nidulans* during microbial confrontations. (A–C) SMBGC maps showing the transcriptional regulation of SM genes in *A. nidulans* during confrontations with *Bacillus amyloliquefaciens* (**A**), *B. subtilis* (**B**), and *Trichothecium roseum* (**C**). Each horizontal track represents one of the 68 predicted SMBGCs in *A. nidulans*. Genes are depicted as arrows indicating strand orientation of genes in the DNA. Colored arrows denote putative functions, with the legend provided at the bottom. Red and blue arrowheads indicate genes with significantly increased or decreased transcript abundance, respectively, during confrontation (adjusted *P* < 0.05). Scale bar represents 10 kb. (**D**) Bar plot showing the number of differentially expressed SM genes with increased (red bars) and decreased (blue bars) transcript abundance across the three confrontations. *A. nidulans* displayed the highest number of differentially expressed SM genes during confrontation with *T. roseum*. (**E**) Venn diagram illustrating the overlap of differentially expressed SM genes in *A. nidulans* during confrontations with the three biocontrol partners. Shared and unique sets of up- and down-regulated SM genes highlight both general and specific responses in secondary metabolism. *PKS*, polyketide synthase; *NRPS*, non-ribosomal peptide synthetase; *PKS-NRPS*, a hybrid of polyketide synthase and non-ribosomal peptide synthetase.

Functional enrichment analysis of GO:BP revealed fundamental differences in the transcriptional responses of *A. nidulans* to the bacterial and fungal antagonists tested in this study. In particular, enriched biological processes pointed to substantial reprogramming of secondary metabolism, suggesting that distinct sets of compounds are likely to be produced in response to each confrontation (Fig. 2). Therefore, we asked how many and which of the 502 SM genes of *A. nidulans* harbored in 68 SMBGCs coordinating synthesis of polyketides (PKs), non-ribosomal peptides (NRPs), hybrid PK-NRPs, indoles, and terpenes (28–30), were differentially regulated in the distinct interactions investigated. Applying the criterion that an SMBGC is considered differentially regulated if at least one of its genes is differentially regulated, only 21 SMBGCs were found to be differentially regulated in *A. nidulans* confronting *B. amyloliquefaciens* (Fig. 3A; Table S8, *An* vs *Ba*). In this confrontation, only the asperfuranone cluster showed increased transcript abundances of all seven genes (Fig. 3A, cluster 57; compare with Fig. 2A). In confrontations with *B. subtilis*, applying the same criterion for differential regulation of a cluster, 40 SMBGCs were found to be differentially regulated in *A. nidulans*, including cluster 57, with all seven genes showing increased transcript abundances (Fig. 3B; Table S8; *An* vs *Bs*). In sharp contrast to the confrontation with *B. amyloliquefaciens*, the interaction with *B. subtilis* led to differential transcriptional regulation of multiple core biosynthetic genes, including those from the fellutamide B cluster 15, the asperlin cluster 32, and the microperfuranone cluster 34. Importantly, 14 out of the 24 genes of the sterigmatocystin cluster 21, also harboring the polyketide synthase gene *stcA*, were transcriptionally increased in the presence of *B. subtilis* (Fig. 3B, SMBGC 21). In addition, eight of the nine genes of cluster 47 required for synthesis of 2,4-dihydroxy-6[(3E,5E,7E)-2-oxonona-3,5,7-trienyl]benzaldehyde were transcriptionally decreased (Fig. 3B, SMBGC 47). Similarly, in cluster 60, where the respective gene products are responsible for the production of 6-ethyl-2,4-dihydroxy-3,5-dimethylbenzaldehyde, six genes were downregulated, while the core biosynthetic gene *pkdA* showed increased transcript abundance (Fig. 3B, SMBGC 60).

Moreover, in hyphae of *A. nidulans* confronting the biocontrol fungus *T. roseum*, data from RNA-Seq analyses suggested pronounced SM responses in *A. nidulans* (Fig. 2C and 3C). In the *A. nidulans* vs *T. roseum* confrontation, 50 of the 68 SMBGCs were differentially transcriptionally regulated (Fig. 3C; Table S8, *An* vs *Tr*), resembling the magnitude of the SM response to *B. subtilis* (Fig. 3B). Intriguingly, in confrontation with *T. roseum*, 19 out of the 50 SMBGCs of *A. nidulans* harbored at least 50% of the transcriptionally differentially regulated genes (Fig. 3C). In clusters 5, 10, 21, 25, 32, 45, and 61, the majority of genes showed increased transcript abundances, with 22 genes of the sterigmatocystin biosynthesis cluster 21 upregulated in this fungus-fungus confrontation. In contrast, the majority of genes harbored in SMBGCs 1, 7, 33, 47, and 60 showed decreased transcript abundances (Fig. 3C).

Collectively, a total of 40, 81, and 79 SM genes were found differentially regulated in *A. nidulans* confronting *B. amyloliquefaciens*, *B. subtilis*, or *T. roseum*, respectively, with 25, 42, and 48 SM genes, respectively, showing increased transcript concentrations in these confrontations (Fig. 3D). These data are in agreement with the primarily altered biological processes, as identified by gene ontology predictions (Fig. 2). Interestingly, only 11 SM genes were commonly differentially regulated in all three interactions, and 11, 32, and 73 genes were exclusively found to be differentially regulated in the *A. nidulans* vs *B. amyloliquefaciens*, the *A. nidulans* vs *B. subtilis*, and the *A. nidulans* vs *T. roseum* confrontations, respectively (Fig. 3E).

RNA-Seq studies have shown differential SM responses in *A. nidulans*, including activation of several genes of the sterigmatocystin cluster (SMBGC 21) by *B. subtilis* and *T. roseum*, but not by *B. amyloliquefaciens* (Fig. 3A through C). As the core *PKS* gene *stcA* (gene accession number ANIA_07825; Fig. S6A) of SMBGC 21 was activated in both the *A. nidulans* vs *B. subtilis* and the *A. nidulans* vs *T. roseum* confrontations, but not in the presence of *B. amyloliquefaciens*, we decided to perform RT-qPCR studies with *stcA*-specific primers (Table S1) to investigate the response of this gene of *A. nidulans* facing the entire set of all bacterial and fungal confrontation partners, as indicated in Fig. 1. To normalize gene expression, the actin gene was used as a reference, with primers given in Table S1. RT-qPCR analyses revealed that among the bacterial confrontations tested, only *B. subtilis* led to an approx fourfold and statistically significant increase in *stcA* transcript levels, as compared to the solo-culture control (*P* < 0.05) (Fig. S6B). Like the other bacterial biocontrol strains, the yeast *S. cerevisiae* did not provoke a statistically significant *stcA* response. In contrast, all filamentous fungi employed as confrontation partners, i.e., *A. tenuis*, *C. heterostrophus*, *C. higginsianum*, *T. roseum,* and *C. domesticus,* elicited highly significant (*P* < 0.001) increases in *stcA* transcript abundances. Importantly, in confrontation with *T. roseum* and *A. tenuis*, *stcA* transcript abundances were increased 25- and 33-fold, as compared to the transcript abundance in hyphae of *A. nidulans* growing in solo culture. Also, in the presence of the biocontrol fungi *T. asperellum* and *T. harzianum*, *stcA* transcript abundances were increased ~10- and ~7-fold, respectively (Fig. S6B).

Collectively, these data show that the establishment of microbial confrontations by introducing biocontrol agents into agricultural environments might cause significant changes in SM gene expression not only in target pathogens but also in ubiquitous microorganisms, such as the fungus *A. nidulans*.

### Microbial confrontations trigger acute reprogramming of secondary metabolite biosynthesis in *A. nidulans*

Strong differential regulation of a large number of genes, including SMBGC genes, in microbial confrontations (Fig. 3) does not necessarily imply metabolome alterations, and hence, complementary untargeted analyses of confrontation-related metabolomes are therefore indispensable.

Employing non-targeted LC-MS/MS analyses, we compared all metabolites produced in *A. nidulans* solo cultures and those produced by this fungus in the various confrontations studied here (Fig. 1; Fig. S7; Table S9). In order to increase the stringency of the data set, features detected in *A. nidulans* solo cultures, as well as those present in both solo cultures and confrontation samples, were excluded from further analysis. Thus, the remaining features represent metabolites that are confrontation-specific.

In the confrontations with the biocontrol bacteria, i.e., *B. amyloliquefaciens*, *B. subtilis*, and *B. velezensis*, 568, 579, and 593 novel and confrontation-specific features, respectively, were identified in *A. nidulans*. Features are uniquely characterized by pairs of mass-to-charge (m/z) and retention time (rt) values and can be regarded as putative compounds or fragments thereof. Features annotated to chemical classes are referred to as compounds. In confrontation with the plant pathogens *A. tenuis*, *C. heterostrophus*, and *C. higginsianum*, 557, 712, and 567 newly and confrontation-specific features were identified, respectively. In confrontations with the biocontrol fungi *T. roseum* and the *Cladosporium* strain isolated from a maize leaf, the formation of 808 and 535 novel features was observed. In the confrontation with the killer strain of *S. cerevisiae*, 495 unique features were detected (Fig. S7A). Identification of features specific to *A. nidulans*, uncontaminated by metabolites from the confrontation partners, was technically easily implementable in the case of distance, zone-line, or contact-type confrontations as described above. However, in confrontations with *C. domesticus*, *T. asperellum,* and *T. harzianum*, all of which exhibited overgrowth of *A. nidulans* mycelium, an additional step was required. Metabolome profiles from solo cultures of *A. nidulans* and the respective confrontation partner were analyzed and subtracted from the profiles obtained from the overgrown *A. nidulans* samples. Following this subtractive filtering approach, 376, 216, and 368 novel features were identified in confrontations of *A. nidulans* with *C. domesticus*, *T. asperellum*, and *T. harzianum*, respectively. The relatively low number of confrontation-specific features observed in overgrowth confrontations may be due to the subtraction of metabolites detected in the pure cultures of *A. nidulans* and its confrontation partners. While these remaining features were attributed to *A. nidulans*, it is important to emphasize the possibility that some may actually originate from the confrontation partner, especially if new metabolites were produced specifically during the interaction within the overgrown regions (Fig. 1A; Fig. S7A).

Applying the software tool Sirius 5.0 (including Canopus and Zodiac; https://bio.informatik.uni-jena.de/software/canopus/) and the chemical ontology system ClassyFire (57) allowed the annotation of less than half of the features identified by non-targeted LC-MS/MS analyses to chemical compound superclasses and classes, despite the fact that the following features were available for annotation: (i), an advanced in-house library of analytical standards and known metabolites, (ii) the KNApSAcK family (35), and (iii) on spectral similarity to the databases NIST17, WEIZMASS (58), Sumner Spectral library (https://www.bruker.com/de/products-and-solutions/mass-spectrometry/ms-software/metabolomics-spectral-libraries.html), MoNA (https://mona.fiehnlab.ucdavis.edu/), GNPS (https://gnps.ucsd.edu/), ReSpect (59) and an in-house database via the spectral library search function of MetaboScape were available for feature annotation. The relatively low number of confrontation-specific features (Fig. S7B) that have been annotated, in spite of the currently available advanced tools, indicates the huge repertoire of novel compounds formed in microbial confrontations. Remarkably, more than half of the detected metabolites could not be assigned to chemical superclasses or classes and are therefore regarded as unknown compounds (Fig. S7B).

To predict compound classes, software framework Sirius (v5.8.6.) with the integrated tools CSI:FingerID, CANOPUS, and ZODIAC, as well as *De Novo* Sum formula predication with Sirius, was used. Chemical class probabilities calculated by the software Sirius/CANOPUS (57, 60) are derived from a supervised machine-learning framework that infers chemical class membership directly from high-resolution tandem mass spectrometry (MS/MS) data. Based on patterns of fragmented patterns, molecular fingerprints yield a spectrum-derived description of molecular structures. CANOPUS uses a large collection of structurally annotated molecules whose class assignments are defined according to a hierarchical chemical ontology (e.g., ClassyFire), and fingerprint feature patterns are associated with chemical classes across multiple ontology levels (superclass, class, subclass). A class probability close to one is a strong indicator of correct chemical class assignment (61), and our own lab experience showed that probabilities above 0.9 usually matched our manual annotation for the chemical class. Indeed, four out of the eight putative azoles exhibit probabilities exceeding a value of 0.9, and this also holds true for three triazines and one quinoline (Table S10). Comparable approaches, including fragment analyses, have been taken previously (see (16). Future analyses should address structural details of these compounds.

**Fig 4:**
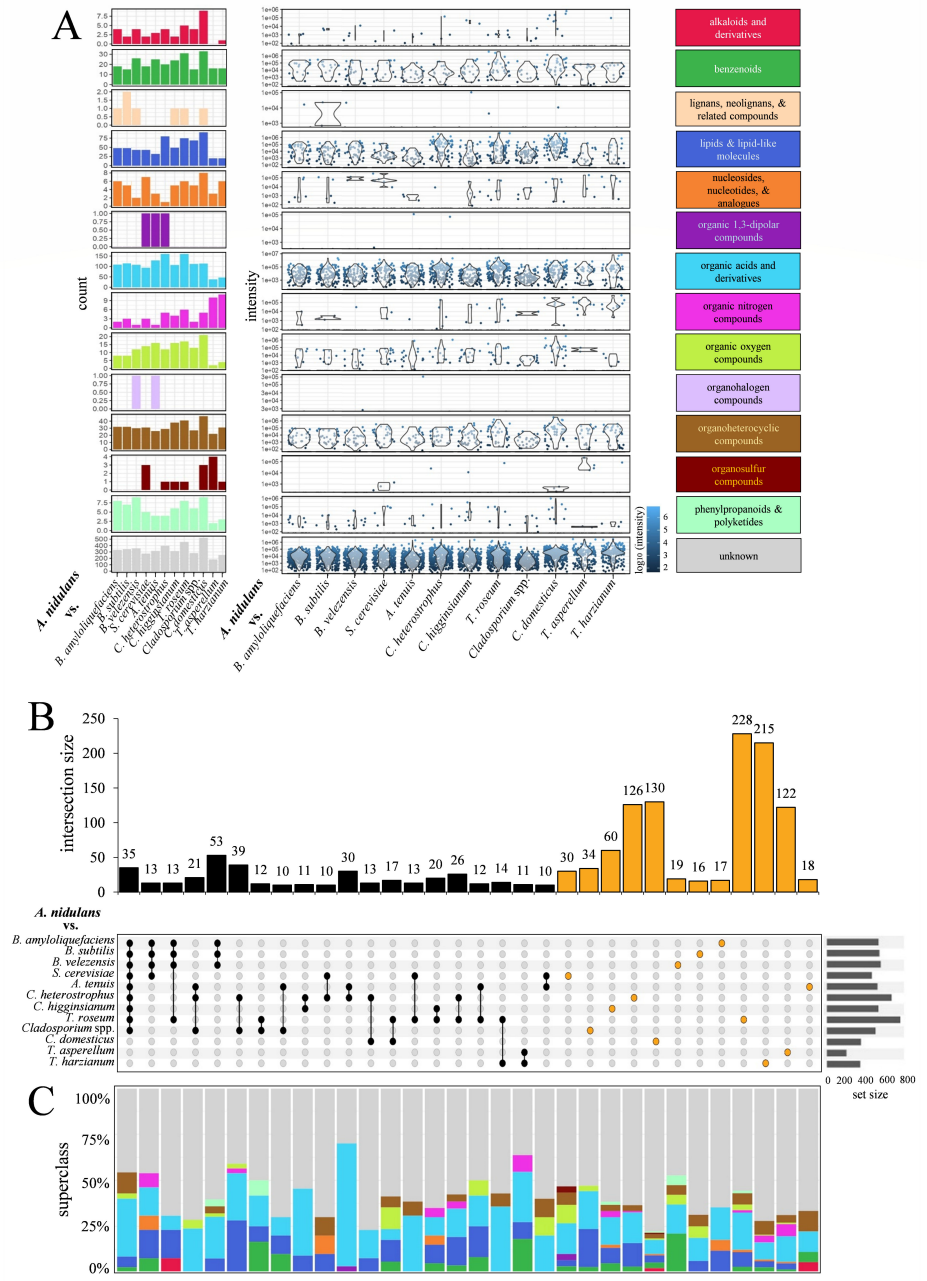
Unique and shared SMs produced by *A. nidulans* under microbial confrontations. (**A**) Classification and intensity distribution of SM features identified across various confrontation conditions. Left panel: bar plots showing the number of SM features assigned to specific superclasses based on ClassyFire annotations, highlighting chemical diversity induced under each interaction. Middle panel: violin plots showing the intensity distribution of individual features, with each dot representing a feature detected in confrontation extracts. Right panel: color key representing the superclasses of the identified features, corresponding to those shown in the left panel. (**B**) UpSet plot illustrating the intersection size and specificity of SM features across confrontation conditions. Upper panel: bars show the number of compounds shared (black bars) between or unique (yellow bars) to specific sets. Lower panel: matrix displays the combinations of confrontation conditions contributing to each intersection. Orange dots represent features unique to a given confrontation, while black dots indicate shared features. The right bar plot shows the total number of detected features per confrontation. Only intersections with ≥10 compounds are shown. (**C**) Stacked bar plots showing the proportional distribution of compound superclasses within the intersections displayed in panel **B**, revealing chemical trends in shared versus unique metabolomes. Colors used in panel **C** correspond to the superclass color key shown in the right panel of **A**.

The annotated confrontation-specific compounds synthesized in *A. nidulans* were assigned to several chemical superclasses, the most prominent of which are organic acids and derivatives thereof, with approx 100 compounds in each confrontation, and lipids and lipid-like molecules, with 25 to 75 confrontation-specific compounds of a wide range of intensities detected in most confrontations (Fig. 4A, left and right panels). Interestingly, confrontation-specific compounds belonging to the SM superclasses of benzenoids and organoheterocyclic compounds were found in *A. nidulans* hyphae confronting any of the tested bacteria or fungi, with count numbers of up to more than 30 and close to 50, respectively, depending on the confrontation, and with strongly varying intensities, as indicated by violin plots (Fig. 4A, middle panel). Fewer compound counts were recorded for the SM superclasses of phenylpropanoids and polyketides, as well as for the alkaloids and derivatives of these. Details of compounds of different classes produced in different confrontations, along with the class probabilities, are given in Table S10. In confrontations with the two *Trichoderma* species, compounds belonging to these superclasses appear to be sparse or missing. However, as in these confrontations, the colonies of *A. nidulans* were overgrown by the *Trichoderma* species, and as these species are known to produce phenylpropanoids and polyketides, as well as the alkaloids (62, 63), compounds of these superclasses may not have been classified as confrontation-specific and were excluded. Again, a large fraction of these features remains unknown (Fig. 4A; compare with Fig. S7B).

Depicting metabolome data in an upset plot further revealed the heterogeneity of the metabolomic responses of *A. nidulans* in various confrontations (Fig. 4B). In some distinct confrontations, identical sets of features were newly synthesized. For example, 35 identical features (Fig. 4B, upper panel) were newly formed in *A. nidulans* confronting all three biocontrol bacteria, the yeast *S. cerevisiae*, and the filamentous fungi *A. tenuis*, *C. heterostrophus*, *C. higginsianum*, *T. roseum*, as well as the *Cladosporium* isolate (Fig. 4B, lower panel, closed circles). These features were annotated as members of five chemical superclasses, e.g., the benzenoids, organoheterocyclic compounds, organic oxygen compounds, organic acids and derivatives thereof, as well as lipids and lipid-like compounds (Fig. 4C, for color codes, see Fig. 4A, right column). In four confrontations, i.e., those including all three bacterial species and *T. roseum*, 13 identical features belonging to the classes of organic acids and derivatives, lipids and lipid-like compounds, as well as alkaloids and derivatives thereof (Fig. 4B and C), and in four confrontations, including those with the three bacterial species and the yeast *S. cerevisiae*, 13 identical features belonging to organic nitrogen compounds, organic acids and derivatives, nucleosides, nucleotides and analogs, lipids and lipid-like molecules, as well as benzenoids were detected (Fig. 4B and C). Large numbers of confrontation-specific compounds were synthesized in single confrontations (Fig. 4B, upper and lower panel; orange circles). For example, 17 compounds were newly found in *A. nidulans* confronting *B. amyloliquefaciens*, 16 were identified in the *B. subtilis* confrontation, and 228 novel compounds, including benzenoids, phenylpropanoids, and polyketides, as well as organoheterocyclic compounds, were identified in *A. nidulans* confronting *T. roseum* (Fig. 4B and C).

**Fig 5:**
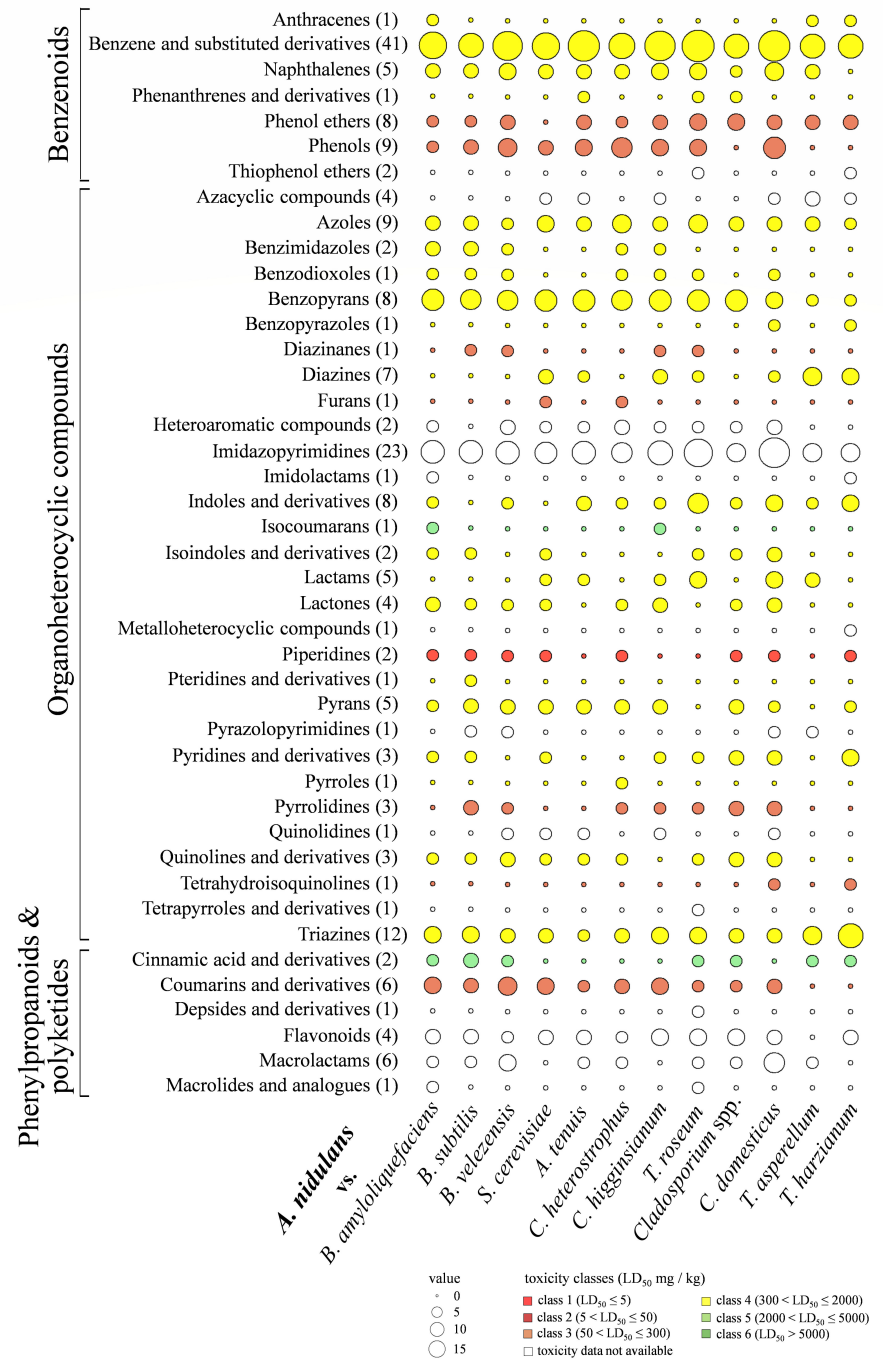
Balloon plot depicting the toxicity of various compound classes in *A. nidulans* under confrontation. The plot depicts the compound classes that are present under three major superclasses, viz., benzenoids, organoheterocyclic compounds, phenylpropanoids, and polyketides. The numbers in parentheses following each compound class indicate the total number of individual compounds present. The acute toxicity of these compounds is color-coded according to their toxicity classes, as indicated by their lethal dose (LD_50_) values. The balloon size indicates the number of compounds present under each confrontation.

As an approximation of the toxicological potential of chemistry classes to mammals, toxicities of their lead structures were subjected to *in silico* toxicity estimations, using ProTox 3.0 (https://tox.charite.de/protox3/index.php?site=home) (64). This tool employs a comprehensive database of approximately 40,000 compounds with known LD_50_ values from rodent experiments (64). Compounds belonging to 43 different chemical classes grouped into the superclasses benzenoids, organoheterocyclic compounds, and phenylpropanoids and polyketides, respectively, were identified in the majority of the confrontations of *A. nidulans*, with benzenoids and derivatives thereof representing the most prominent class (Fig. 5). Two putative piperidines representing toxicity class I compounds, with predicted LD_50_ values of ≤5 mg/kg body weight, were identified in *A. nidulans* confronting all three *Bacillus* species, and also in confrontation with the killer toxin-producing strain of *S. cerevisiae,* as well as with *C. heterostrophus*, *Cladosporium* spp., *C. domesticus,* and *T. harzianum* (Fig. 5). Moreover, several putative phenol ethers, phenols, diazinanes, furans, pyrrolidines, a tetrahydroisoquinoline, as well as coumarins and derivatives thereof, were classified into chemical categories associated with significant toxicity classes. Strikingly, numerous compounds identified, i.e., putative azoles, benzimidazoles, piperidines, quinolones, triazines, as well as cinnamic acid derivatives, belong to chemical classes used as synthetic fungicides (https://ipm.ifas.ufl.edu/resources/success_stories/t&pguide/pdfs/appendices/appendix6-frac.pdf), which may, in part, explain the establishment of distance inhibition in numerous confrontations (Fig. 1). However, as only compound classes rather than individual compounds have been identified, the toxicity classes assigned to these classes may not directly relate to the substances produced in microbial confrontations.

**Fig 6:**
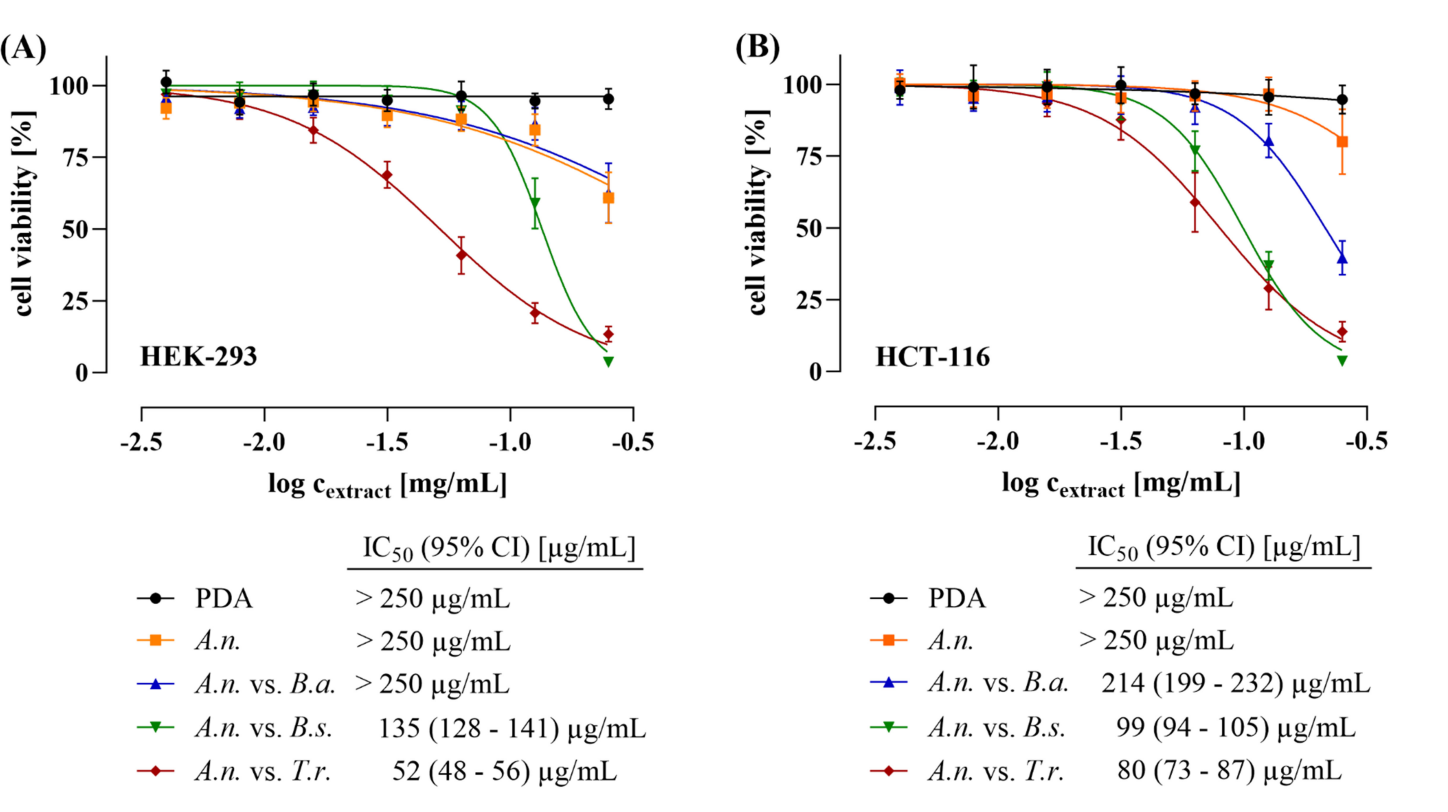
Cytotoxic effects of SMs produced by *A. nidulans* under microbial confrontation on human cell lines. IC_50_ curves representing the effect of crude metabolite extracts from *A. nidulans* grown alone, in confrontation with *Bacillus amyloliquefaciens*, *B. subtilis,* and *Trichothecium roseum*, respectively, on the viability of (**A**) healthy human embryonic kidney cells (HEK-293) and (**B**) human colorectal carcinoma cells (HCT-116). Cells were treated for 48 h, followed by cell viability read-out using the fluorometric resazurin assay. Data were collected from at least four biological replicates, each with technical triplicates. IC_50_ curves and values were calculated using GraphPad Prism v10.1 software. Error bars indicate the 95% confidence interval (CI).

While it is tempting to speculate, based on estimated toxicity classes (Fig. 5), on health risks for consumers of crops treated with biological control agents, predictions may be misleading. Therefore, we extracted compounds from *A. nidulans* confronting the biocontrol agents *B. amyloliquefaciens*, *B. subtilis,* and *T. roseum*. An extract of the non-colonized PDA and an extract of *A. nidulans* growing without a confrontation partner served as controls. All extracts were tested *in vitro* with respect to their impact on the viability and growth of two human cell lines. For that purpose, human embryonic kidney (HEK-293) and colorectal cancer cells (HCT-116) were incubated with the extracts at concentrations ranging from 0.98 to 250 µg/mL. Subsequently, after 48 h of treatment, the viability of the cells was measured by using the resazurin cell viability assay (38, 39). Extracts containing mixtures of metabolites from *A. nidulans* confronting *B. subtilis* or *T. roseum*, respectively, exhibited the most severe toxic effects on both HEK-293 and HCT-116 cells. Forty-eight hours after adding the highest extract concentration, i.e., 250 µg/mL, from the *A. nidulans* confrontations with *B. subtilis* or *T. roseum,* only 3.6% and 13.4% of the HEK-293 cells, and 3.6% and 13.8% of the HCT-116 cells, respectively, remained viable (Fig. 6). IC_50_ values of 135 and 52 µg/mL for HEK-293 cells, and 99 and 80 µg/mL for HCT-116 cells were calculated for the extracts of *A. nidulans* vs *B. subtilis* and *A. nidulans* vs *T. roseum*, respectively. Remarkably, the IC_50_ values of extracts of *A. nidulans* solo cultures were significantly lower, i.e., >250 µg/mL for both cell types. The data indicate that microorganisms synthesize secondary metabolites clearly differing in their acute toxicity levels, depending on the confronting partner(s). As IC_50_ values measured in this study reflect combined toxic activities of complex compound mixtures, i.e., crude extracts (Fig. 6), it is reasonable to assume that individual metabolites contained in these mixtures may have a significant impact on the viability of human cells, as exemplarily shown for healthy human kidney and colorectal cancer cells.

Collectively, using defined pairs of dual confrontations, we show that biosynthesis of the same group of SMs may be triggered by several confrontation partners but that other distinct compounds are formed exclusively and in a confrontation-specific manner (Fig. 4B). In the context of environmental microbial populations, our data suggest that estimates of SM formation and of the toxicological risk imposed by these compounds should be considered in the approval of microbial biocontrol agents in plant protection.

## DISCUSSION

The phyllosphere is densely colonized by microorganisms of various genera (5–7, 65, 66). Incubation of agar medium imprints of maize leaves has shown that the vast majority of microbes grow until they contact neighboring colonies, which ends colony expansion. However, distance inhibition, indicative of secretion of antimicrobial compounds by at least one partner, only rarely occurred (16).

Distance inhibition is widely recognized as a hallmark of antagonistic interactions (41, 67). For instance, confrontation assays between the phytopathogens *Eutypa lata* and *Botryosphaeria obtusa* resulted in the production of antimicrobial compounds belonging to the class of mycoalexins that are involved in fungal defense responses (68). Similarly, in another study involving the basidiomycete *Stereum hirsutum* and its antagonists, *Coprinus micaceus* and *Coprinus disseminatus*, several phenolic SMs were detected, including 1-methyl-3,5-dihydroxybenzene and 1,2-dihydroxyanthraquinone (69). These findings highlight the chemical diversity and ecological relevance of inducible compounds produced during microbial competition. In spite of this chemical diversity, extended screens have to be conducted in order to identify strains showing biological control activity toward plant pathogens (43, 70), since antagonistic microorganisms functioning in natural agricultural environments are very rarely found.

Successful competition is thought to be chemistry-based (67), hence, most microorganisms harbor significant numbers of SMBGCs per genome, with the exception of yeasts (71) and some biotrophic plant pathogens (72–74). For example, in the fungal class of Eurotiomycetes, an average of 48 clusters has been identified per species, and, remarkably, 25% of the species within this class possess more than 60 SMBGCs (9). Bacterial genomes harbor similar numbers of SMBGC (10). These numbers suggest that secondary metabolites of enormous chemical diversity might act as effective modulators in microbial confrontations. However, while most of the SMBGCs are silent in solo cultures, they become activated in confrontations with other microbes in order to increase the producers’ competitiveness for their ecological niches (8, 75–77). Recent dual confrontation experiments employing *Colletotrichum graminicola*, a maize pathogen causing leaf anthracnose and stalk rot (78), and either the biocontrol bacterium *B. amyloliquefaciens* or the fungus *A. nidulans* suggested activation of confrontation partner-specific subsets of SM biosynthetic genes (16). Furthermore, in microbial interactions, the number of compounds formed could be increased significantly, as SMs produced by one species can be taken up and further modified by another, creating a chemical environment of unpredictable toxicity for most exposed organisms (12).

In order to improve our understanding of the specificity of microbial interactions, as well as the complexity of the chemical compounds generated in microbial confrontations, we established 12 interactions involving the ascomycete *A. nidulans*—one of the best-studied fungi in terms of secondary metabolism—and plant pathogenic fungi, fungi isolated from the maize phyllosphere, as well as a selection of bacterial and fungal biocontrol agents. Interestingly, RNA-Seq and gene ontology analyses revealed activation of distinct SM genes and biological processes in *A. nidulans*, suggesting specific antagonistic mechanisms activated in confrontations even with closely related microbes.

Non-targeted LC-MS/MS analyses allowed the identification of newly synthesized compounds, some of which were found in a number of confrontations, while others were confrontation-specific. A striking finding of this study was that compounds formed in several confrontations, i.e., putative azoles, benzimidazoles, piperidines, quinolones, triazines, and cinnamic acid derivatives, share their lead structures with synthetic fungicides. In total, more than 30 compounds falling into these chemical classes have been identified in the *A. nidulans* confrontations described here.

One may argue that although *A. nidulans* is a powerful model organism in the analysis of secondary metabolism, the generalization of its confrontation responses in an agricultural context should be made cautiously. However, a previous study addressing the transcriptional and metabolomic response of a plant pathogenic fungus, i.e., the maize anthracnose fungus *C. graminicola*, also revealed altered expression of an enormous number of SM genes in confrontations with the biocontrol bacterium *B. amyloliquefaciens* and with *A. nidulans*. Interestingly, in these confrontations, putative azoles, piperidines, and cinnamaldehydes were also synthesized (16). Thus, although the similarities in the chemical response of these distinct confrontations may not allow us to draw a general conclusion, these data strengthen the observation that microbial defense against competitors is primarily based on the synthesis of toxic chemicals (67), and that some of the compounds produced in microbial confrontations structurally resemble synthetic fungicides.

As stated above, confrontation-mediated changes in the expression of SM genes and SMs reported in this study are correlative. It would be interesting to strengthen the causal links between changes in transcriptional responses to confrontation partners and the formation of compounds. To accomplish this goal, future studies could employ targeted mutagenesis of individual SM genes or SMBGCs and analyses of the resulting mutants in confrontation assays and SM production in response to competitors. Alternatively, to provide a first indication of the general importance of SM production in microbial competitiveness, mutants lacking 4′-phosphopantetheinyltransferase, which are unable to post-translationally activate the entire set of all polyketide synthases and all non-ribosomal peptide synthetases (79, 80), could be generated and employed in confrontation assays.

It is surprising to note that chemical structures similar to those discovered in microbial confrontations have also been identified in industrial fungicide screens. Interestingly, not only one, but rather several compounds belonging to different chemical classes were formed in each confrontation in order to address distinct targets and to inhibit fungal growth and development. It is likely that the synthesis of a cocktail of putatively antifungal compounds may not only strongly increase the inhibitory efficacy but also avoid the development of resistance to such compounds driven by mutational changes in the molecular targets of single substances. Indeed, a large body of literature shows that in several plant pathogenic fungi, mutation-based amino acid exchanges, e.g., the G143A exchange in cytochrome *b*, the E198A/V/K substitutions in β-tubulin, or the L143F+G446S double mutations in cyp51, conferring resistance to strobilurin, benzimidazole, or azole fungicides, respectively, cause failure in chemical disease control (81–84). However, as mutations in those genes causing alterations of fungicide target sites are rather rare (85), simultaneous occurrence of double or triple mutations in a single conidium would be extremely unlikely and thus be of minor concern. Therefore, in agricultural environments, production of a mixture of putatively antifungal compounds by antagonistic microorganisms would counteract the advantages of plant pathogens with an amino acid exchange at a single target site of a synthetic fungicide. Intriguingly, in conventional plant protection, the availability of three fungicides with distinct modes of action is thought to be required to counteract the development of fungicide resistance (2), and references therein. Based on these considerations, one may hypothesize that mutants of plant pathogens carrying only single amino acid exchanges in fungicide target sites would be unlikely to show reduced sensitivity to antagonists synthesizing mixtures of the above-mentioned antifungals. On the other hand, it would be interesting to test the efficacy of antagonists producing an array of antifungal compounds in confrontations with fungicide-adapted strains, which often show significant activities of multi-specific efflux transporters, simultaneously affecting the efficacies of several drugs (86).

In this study, we show that a large array of compounds is produced in *A. nidulans* when confronting antagonistic microorganisms. However, although these findings appear to support the use of antagonistic microorganisms in plant disease control, it is important to consider that the majority of the individual compounds produced in different confrontations are structurally and toxicologically unknown. An indirect estimate of the toxicities of some identified compounds and the even more demanding identification of compound mixtures, by *in silico* toxicity estimations (see above), is an insufficient approach to assess consumer safety. Therefore, we have decided to perform *in vitro* toxicity assays employing human colorectal carcinoma and human embryonic kidney cells, whereby tissues of both of which are likely to come into contact with ingested microbial SMs synthesized in antagonist-treated crops. Assuming that only a few of the large array of compounds synthesized would be toxic, we were surprised to discover low IC_50_ values below 100 µg/mL for extracts, i.e., mixtures of the synthesized SMs, produced as a consequence of the confrontation between *A. nidulans* with *B. subtilis* and *T. roseum*, respectively, with both human cell types. As these mixtures of compounds tested displayed critical toxicity, one may assume that some of these SMs might be even more toxic to these human cell lines. In order to address the fact that *in vitro* toxicity assays, as employed in this study, might not reflect the situation of food derived from plants treated with antagonistic microorganisms, future studies should include tests mimicking food processing of the extracts containing putatively toxic SMs.

A webinar held at the British Society for Plant Pathology and the UN initiative “International Year of Plant Health 2020” stated that risks associated with the use of antagonistic microorganisms are not to be expected and highlighted the fact that legislation and registration of antagonists are comparable to synthetic pesticides (87). At first glance, this view may reduce concerns regarding the toxicity of compounds synthesized by antagonists. However, we have demonstrated that the toxicity of novel SMs specifically synthesized in response to interactions and mixtures thereof, produced by *A. nidulans* confronting *B. subtilis* or *T. roseum,* clearly exceeds that produced in solo culture. Moreover, our findings strongly indicate that large numbers of different compounds with unknown structural and toxicological properties might be expected in multiple natural microbial interactions and in antagonist-treated agricultural environments. Hence, realistic consumer risk assessments might be far more complex and may be barely feasible to conduct effectively. The fact that unknown confrontation-specific compounds are not taken into account during legislation and registration of antagonists, despite their putative toxicity, requires a paradigm shift in legislation and application of microbial antagonists.

These concerns outlined above do not *per se* argue against using antagonistic microorganisms in crop protection and may be circumvented by employing microorganisms that do not primarily rely on toxic SMs in confrontations. A distinct concept in antagonist-based plant protection may be deduced from a complex screen of determinants of bacterial interactions in the phyllosphere microbiota, using a 15-member synthetic community to which 200 endogenous bacterial strains were added, and subsequent changes in the community composition were recorded. This systematic *in planta* screen identified an *Aeromicrobium* strain that caused lysis of cells of another phyllosphere-inhabiting bacterium, a member of the *Actinomycete* genus *Nocardioides*. Ethyl methanesulfonate-induced mutagenesis of *Aeromicrobium* and a loss-of-lysis screen identified an S70L mutation in an endo-cleaving peptidase possibly involved in cleavage of peptidoglycan cross-linkages of the bacterial envelope (88). Though the *Aeromicrobium* endopeptidase may play a role in community assembly on *Arabidopsis* leaves, it is perhaps doubtful that strains secreting these enzymes would effectively antagonize plant pathogenic bacteria, as the target range of bacteriolytic enzymes, including the *Aeromicrobium* endopeptidase, is taxonomically narrow, leaving fungal target pathogens putatively unaffected (88). However, bacterial genomes harbor a plethora of SMBGCs (10), and although the role of an endopeptidase in *Aeromicrobium* confronting *Nocardioides* species has been convincingly demonstrated (88), it would be interesting to investigate how many novel secondary metabolites are synthesized in the interaction between these phylloplane bacteria, in addition to endopeptidase formation.

To develop a consumer-safety strategy in plant protection, identifying or generating microorganisms lacking or harboring a minimum number of SMBGCs is required, as is the case in biotrophic plant pathogens like the maize smut fungus *Ustilago maydis* (74). In contrast to the idea that effectors are exclusively required to compromise host defense, recent studies have shown that some effector genes are expressed at pre-invasion development on the host surface (89). Recently, Doehlemann et al. showed that the effector ribotoxin Ribo1, a secreted ribonuclease, cleaves the widely conserved sarcin-ricin loop of rRNA, interferes with protein biosynthesis, and thus causes death of a wider range of interacting microorganisms. The role of Ribo1 in enhancing the competitiveness of the maize smut fungus on the plant surface was supported by the observation that a Ribo1-deficient mutant was less effective at competing with biocontrol bacteria compared to the Ribo1-expressing wild-type strain (90). Accordingly, a Ribo1-producing strain defective in factors required for plant invasion and pathogenicity, such as the effector proteins Pep1 (protein essential for penetration), or Rsp3 (repetitive secreted protein 3) (91), would produce Ribo-1 on the plant surface but fail to cause disease and would thus be suited to inactivate leaf pathogens without forming putatively toxic SMs.

Taken together, our findings underscore the remarkable chemical diversity and biological potency arising from microbial confrontations, particularly in the context of secondary metabolite production by *A. nidulans*. In spite of the fact that *A. nidulans* may be of limited relevance in agricultural settings and although potential consumers’ SM exposure levels are largely unknown, the discovery of toxicity to human cells of SM mixtures produced during confrontations, which are often absent in solo cultures, highlights a critical blind spot in current legislation governing microbial biocontrol agents. These insights call for confrontation-informed risk assessment protocols. At the same time, our data suggest the feasibility of developing innovative, effector-based biocontrol strategies, as exemplified by *U. maydis* Ribo1 (90), which minimize reliance on chemically undefined SMs. By integrating both metabolic potential and targeted molecular approaches, future antagonist design can move toward a safer, more predictable, and ecologically sound paradigm in microbial crop protection.

## SUPPLEMENTAL MATERIAL

Supplemental figures aem.02180-25-s0001.docx Fig. S1 to S7.

Supplemental tables aem.02180-25-s0002.xlsx Tables S1 to S10.

ASM does not own the copyrights to Supplemental Material that may be linked to, or accessed through, an article. The authors have granted ASM a non-exclusive, world-wide license to publish the Supplemental Material files. Please contact the corresponding author directly for reuse.
