# Supplementary material for: Confrontations between Aspergillus nidulans and microbial biocontrol agents cause differential regulation of secondary metabolism and synthesis of chemicals toxic to human kidney and colon cells
Source: Appl Environ Microbiol. 2026 Apr 13;92(5):e02180-25. doi: 10.1128/aem.02180-25 (PMC13188921; doi:10.1128/aem.02180-25)
Supplement: Supplemental figures — Fig. S1 to S7. [file aem.02180-25-s0001.docx]

Supplemental Figures

Confrontations between *Aspergillus nidulans* and microbial biocontrol agents cause differential regulation of secondary metabolism and synthesis of chemicals toxic to human kidney and colon cells

Bennet Rohan Fernando Devasahayam^1,2^, Yvonne Poeschl^3,4^, Henriette Uthe^2,5^, Robert Rennert^5^, Lena Hartmann^1^, Holger B. Deising^1,3,*^

^1^ Faculty of Nutritional Sciences III, Chair for Phytopathology and Plant Protection, Martin Luther University Halle-Wittenberg, Institute of Agricultural and Nutritional Sciences, Halle, Germany

^2^ EcoMetEoR, Molecular Interaction Ecology, German Center for Integrative Biodiversity Research (iDiv), Leipzig, Germany

^3^ German Center for Integrative Biodiversity Research (iDiv), Leipzig, Germany

^4^ Biometrics and Agricultural Informatics, Faculty of Natural Sciences III, Martin Luther University Halle-Wittenberg, Halle, Germany

^5^ Department of Bioorganic Chemistry, Leibniz Institute of Plant Biochemistry, Halle, Germany


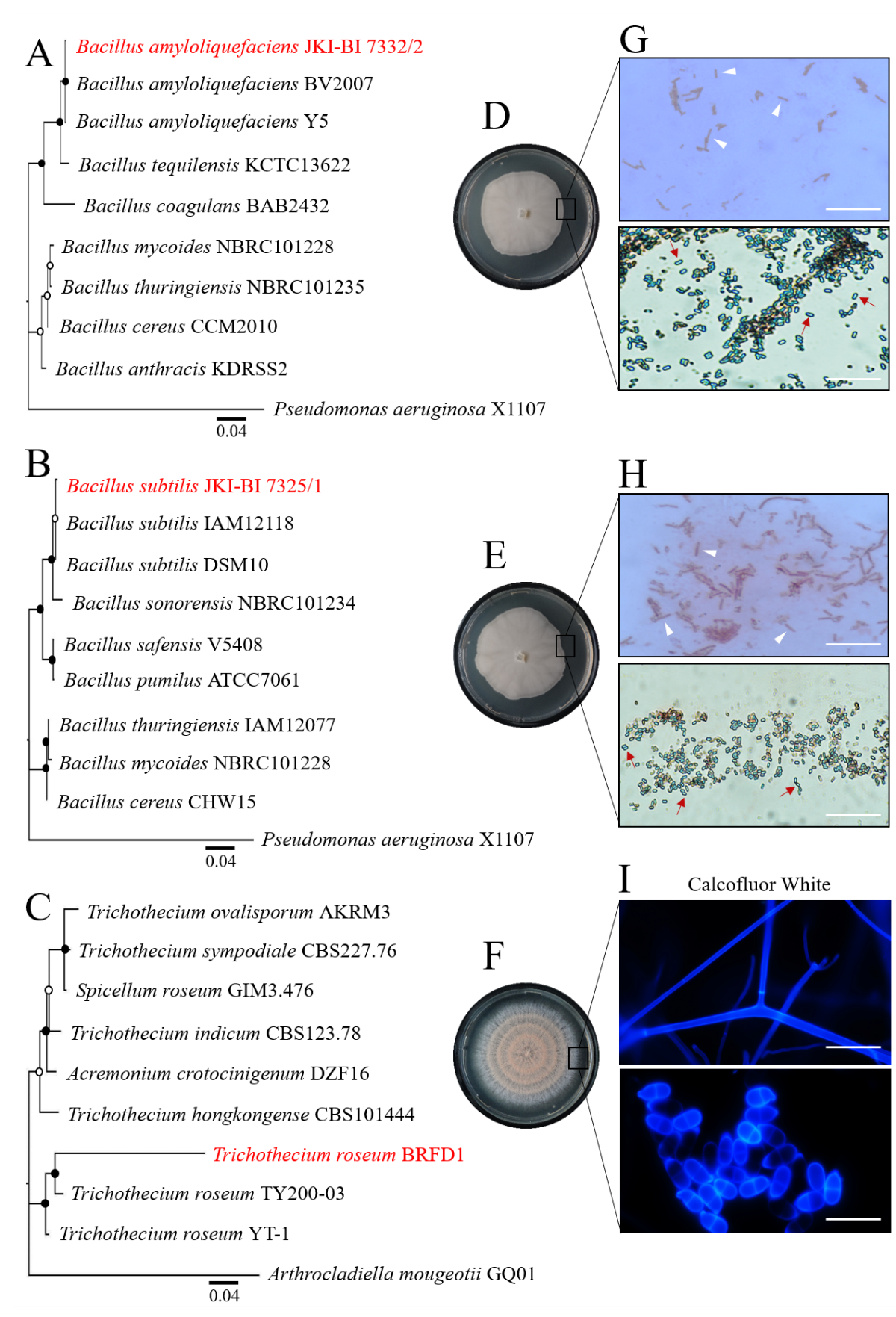


**Supplemental Figure S1. Phylogeny and morphological characterization of bacterial** **and fungal biocontrol strains employed in transcriptome assays.**

**(A** and **B)** shows the phylogenetic trees based on 16S rRNA gene sequences of *B. amyloliquefaciens* JKI-BI 7332/2 and *B. subtilis* JKI-BI 7325/1, showing the close relationship of *B. amyloliquefaciens* JKI-BI 7332/2 to other *Bacillus* species. The outgroup was assigned to *P. aeruginosa*.

**(D, E, G** and **H)** shows the morphological characterization of *B. amyloliquefaciens* and *B. subtilis* indicating the colony morphology on PDA 4dpi, while the microscopy panel shows a Schaeffer Fulton stain of the bacterial cells, highlighting the presence of vegetative cells (stained red and indicated by white arrowheads) and endospores (stained green and indicated by red arrows). Scale bars represent 50 µm.

**(C)** Phylogenetic tree based on ITS region sequences of *T. roseum* BRFD1, showing

its relationship with other *Trichothecium* species. *A. mougeotii* GQ01 is assigned as an outgroup.

**(F)** Morphological characterization of *T. roseum* with colony morphology shown on a Petri dish with PDA at 7 dpi.

**(I)** The microscopy panel shows the calcofluor white-staining of hyphae (upper) and spores (lower), highlighting the chitin-rich cell walls. Scale bars represent 50 µm.

**(A, B,** and **C)** The Bootstrap values are indicated by circles, with filled circles representing higher (> 0.9) and open circles representing lower support (< 0.9). Scale bar of phylogenetic tree represents 0.04 substitutions per site.


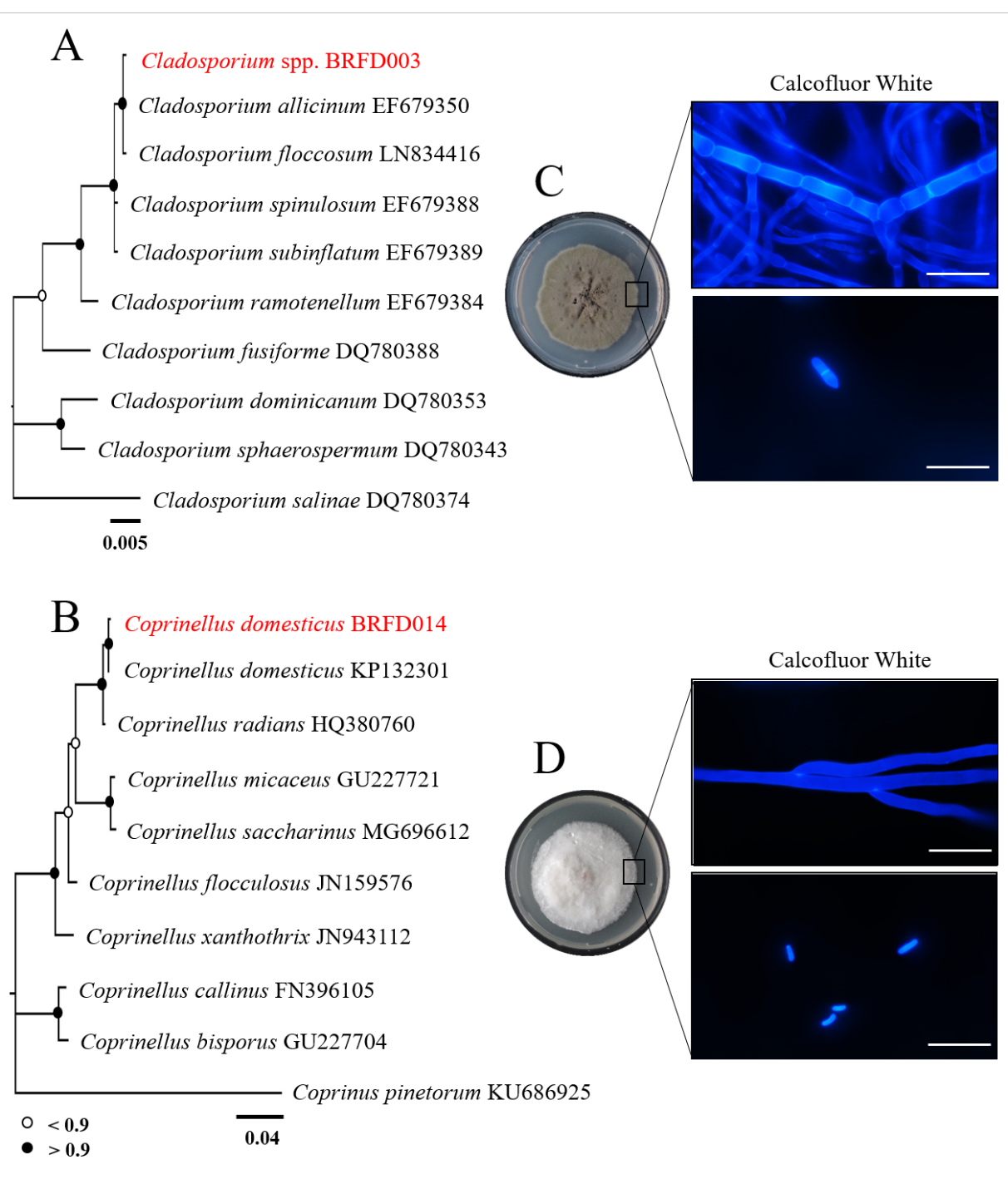


**Supplemental Figure S2. Phylogenetic identification and morphological characterization of fungal isolates from maize phyllosphere.**

**(A)** Maximum likelihood phylogenetic tree based on ITS sequences showing the placement of *Cladosporium* spp. BRFD003. The isolate clusters closely with *Cladosporium allicinum* EF679350 and *Cladosporium floccosum* LN834416, indicating high sequence similarity. *Cladosporium salinae* DQ780374 was used as the outgroup. Scale bar represents 0.005 substitutions per site.

**(B)** Phylogenetic tree of *Coprinellus domesticus* BRFD014, constructed using ITS sequences. The isolate shows high similarity to *Coprinellus domesticus* KP132301, with strong bootstrap support. *Coprinus pinetorum* KU686925 was used as the outgroup. Scale bar indicates 0.04 substitutions per site.

**(A** and **B)** Filled and open circles at nodes indicate bootstrap values > 0.9 and < 0.9, respectively.

**(C)** Colony morphology of *Cladosporium* spp. BRFD003 grown on PDA, forming a dense, greenish-gray, fluffy mycelium at 7 dpi.

**(D)** Colony morphology of *Coprinellus domesticus* BRFD014 on PDA showing white, cottony, and velvety growth at 7 dpi.

**(C** and **D)** Fluorescence microscopy images adjacent to each colony show Calcofluor white-stained hyphae (upper panels) and spores (lower panels), revealing fungal cell wall structures. Scale bars represent 50 µm.


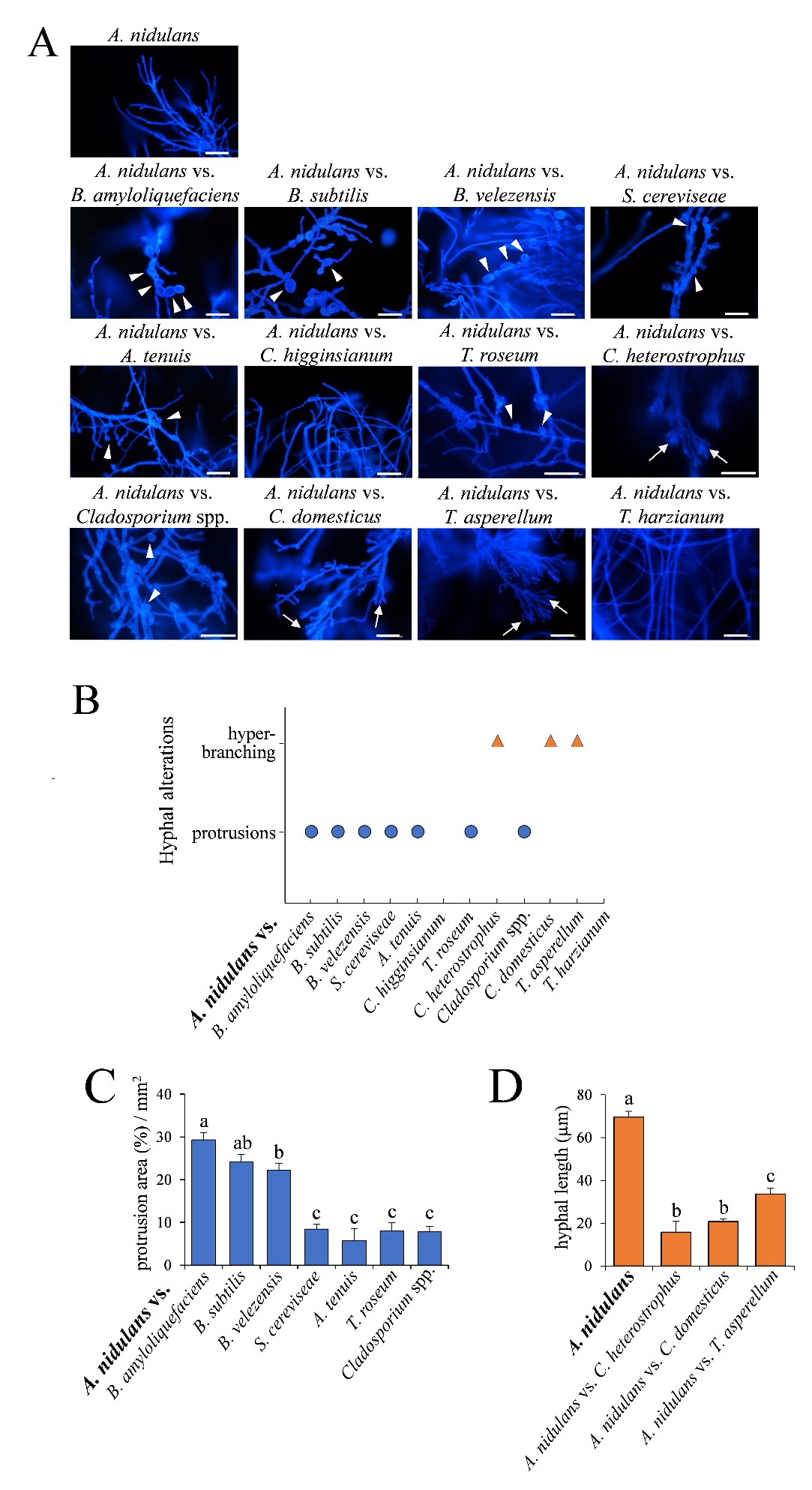


**Supplemental Figure S3. Microscopic visualization and quantitative assessment of hyphal deformations in *A. nidulans* under microbial confrontations.**

**(A)** Fluorescence microscopy images of *A. nidulans* hyphae stained with Calcofluor White reveal morphological alterations during microbial confrontation at 14 days post-inoculation (dpi). Prominent hyphal swellings were observed in confrontations with biocontrol *Bacillus* spp. (white arrowheads), while fungal confrontations induced mild hyphal protrusions as lateral protuberances (white arrowheads) and excessive hyper-branching (white arrows). Scale bar are 50 µm.

**(B)** Quantification of hyphal modifications, including protrusions (blue circles) and hyper-branching (orange triangles), observed in *A. nidulans* during confrontations with different microbial partners.

**(C)** Comparative analysis of protrusion area percentages demonstrates a significant increase in protrusion formation in *A. nidulans* during *Bacillus* confrontations relative to fungal counterparts.

**(D)** Measurement of hyphal branching lengths in deformed hyphae of *A. nidulans* reveals a marked reduction in branch length during microbial interaction compared to solo-culture.

All experiments were conducted in triplicates. Error bars in (C) and (D) indicate standard deviations (+ SD). Distinct letters (a, b, c) indicate statistically significant differences between treatment means as determined by Tukey’s Honest Significant Difference (HSD) test (*p* < 0.05), where different letters denote statistically distinct groups.


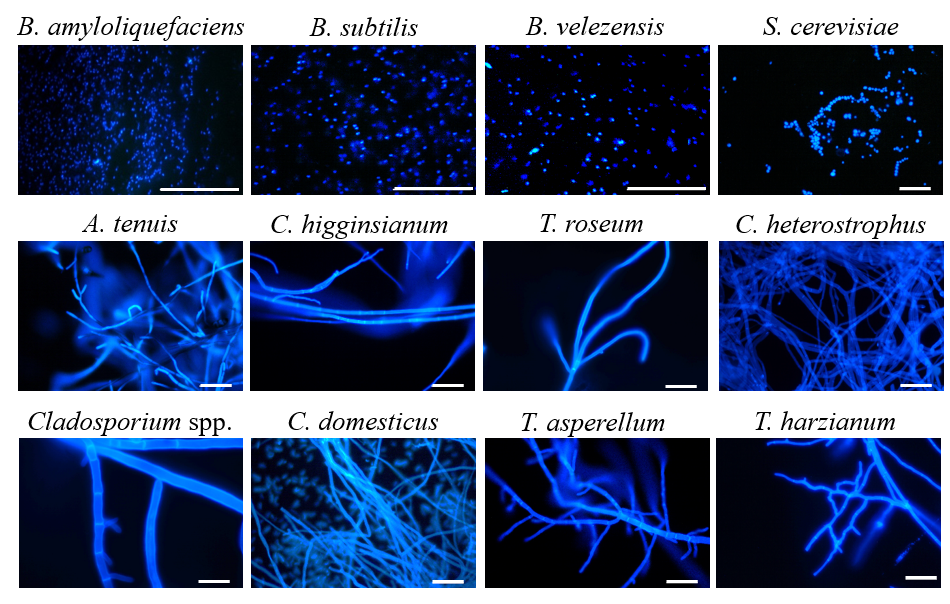


**Supplemental Figure S4. Fluorescence microscopy of fungal confrontation partners used as controls.**

Calcofluor white-stained hyphae and spores of the microbial species used in confrontation assays with *A. nidulans*, imaged under fluorescence microscopy to serve as structural controls. The top panel shows the *Bacillus* or *S. cerevisiae* cells, while the middle and the lower panels display the hyphal morphology of each respective fungal confrontation partner. These images represent the native hyphal architecture of the antagonistic microbes in monoculture, enabling comparison with altered hyphal phenotypes of *A. nidulans* shown in Supplemental Figure S3A. Scale bars represent 50 µm.


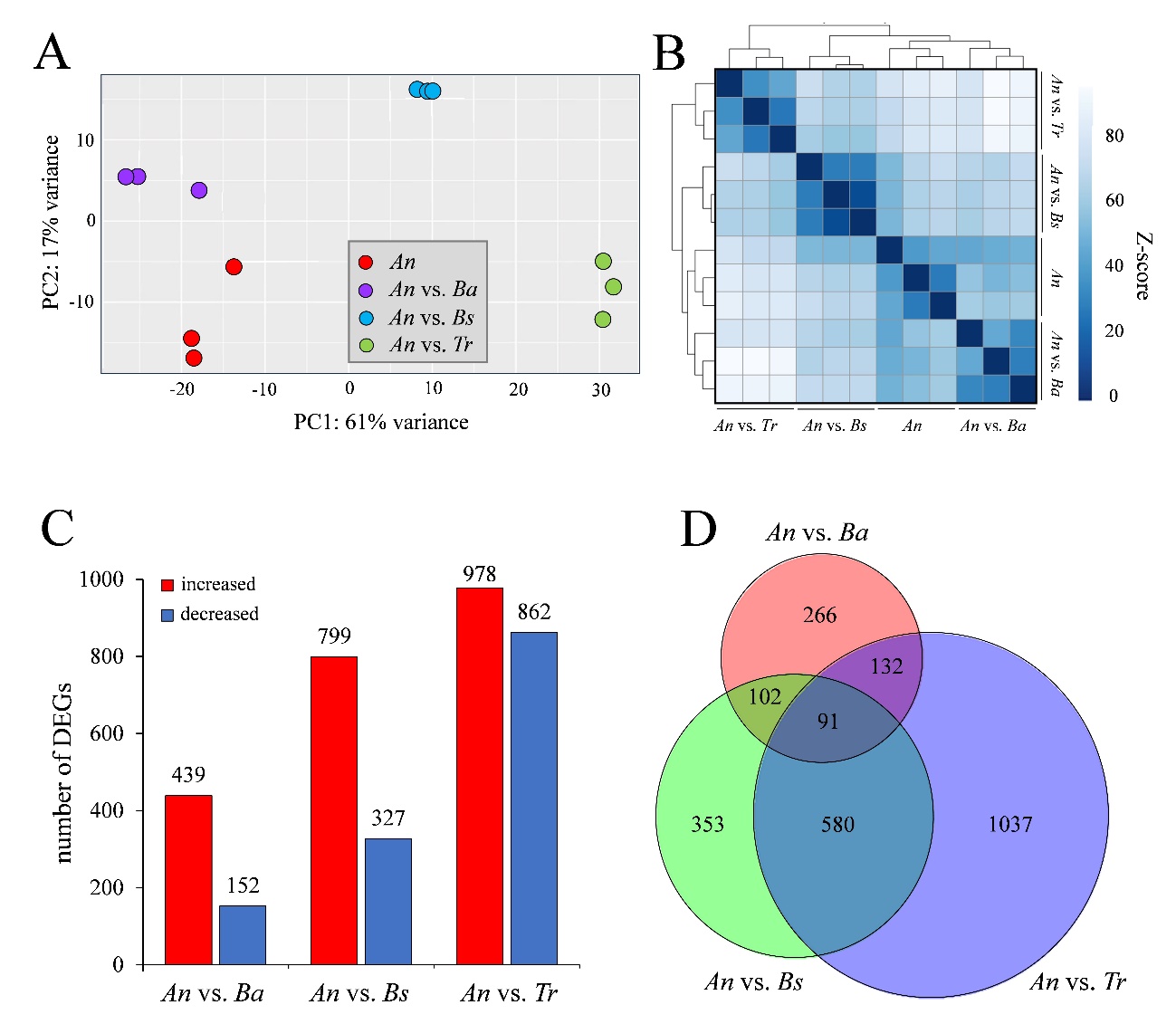


**Supplemental Figure S5. Transcriptome analysis of differentially expressed genes (DEGs) of *A. nidulans* under confrontations.**

**(A)** Principal Component Analyses (PCA) show clear cluster separation of *A. nidulans* solo-culture (*An*) and hyphae confronting *B. amyloliquefaciens* (*An* vs. *Ba*), *B. subtilis* (*An* vs. *Bs*), and *T. roseum* (*An* vs. *Tr*). Sample groups are indicated by different color codes. Each replicate is plotted as an individual data point. Experiments were performed in triplicate.

**(B)** Heatmap of the sample-to-sample distance matrix obtained from solo-culture of *A. nidulans* and confrontations with *B. amyloliquefaciens, B. subtilis*, and *T. roseum*. The color codes indicate the distance between the samples, based on the Z-score. Dark blue denotes shorter distance, i.e., replicates are grouped closer to each other.

**(C)** Bar plot indicates the DEGs identified in different confrontations. Red and blue bars indicate increased (FC > 2) and decreased (FC < 0.5) transcript abundances with adjusted *p* < 0.05.

**(D)** Venn diagram representing the distribution of the DEGs of *A. nidulans* confronting *B. amyloliquefaciens* (*An* vs. *Ba*), *B. subtilis* (*An* vs. *Bs*), and *T. roseum* (*An* vs. *Tr*). The numbers in the overlaps denote the mutual DEGs between distinct confrontations.


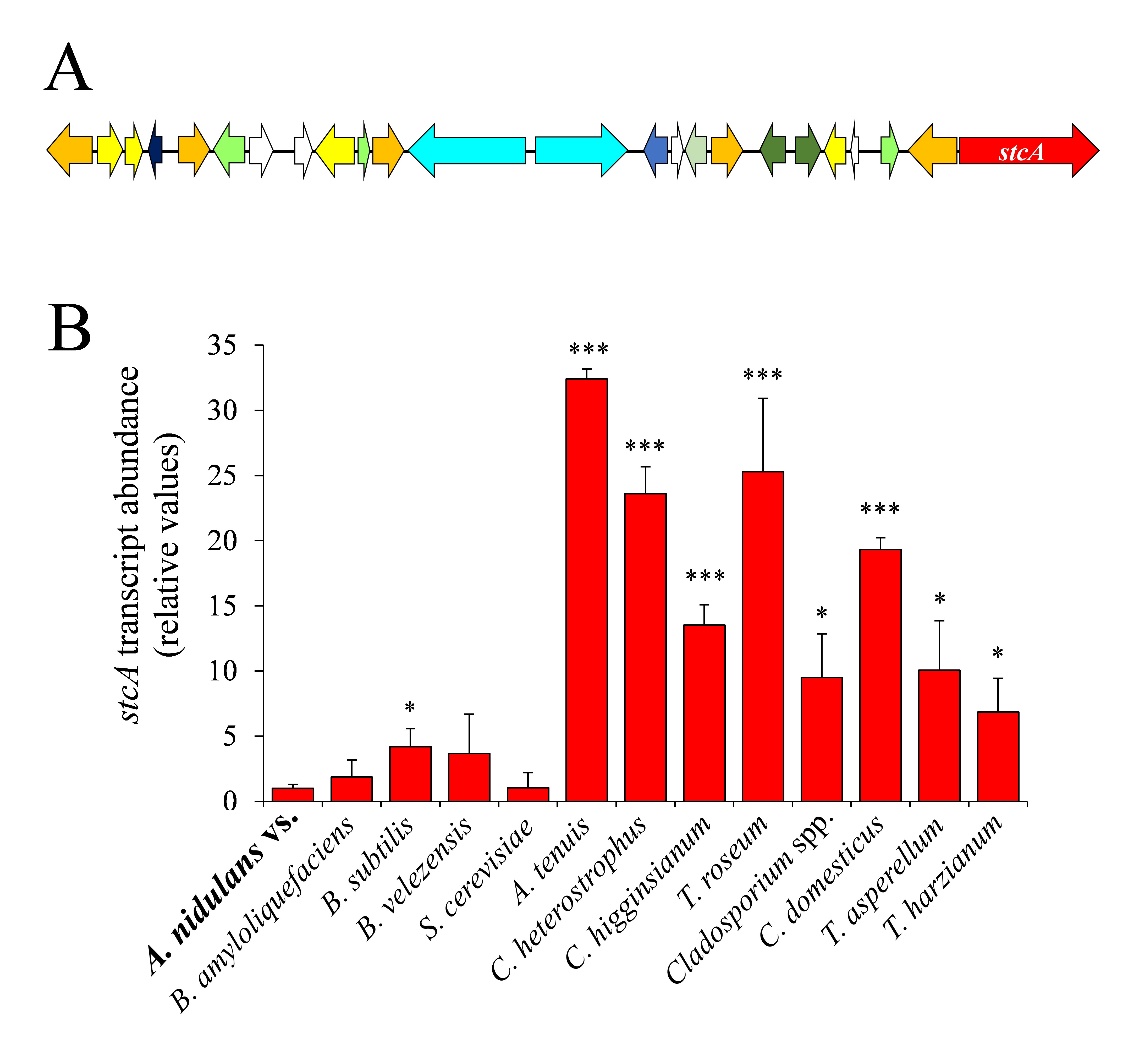


**Supplemental Figure S6. Quantitative expression analysis of the *stcA* gene in *A. nidulans* during microbial confrontations.**

The transcript abundance of *stcA*, the core *PKS* gene of the sterigmatocystin (STC) biosynthetic gene cluster (SMBGC 21), was assessed by RT-qPCR in *A. nidulans* during confrontation with various bacterial and fungal species.

**(A)** The physical map of SMBGC 21 is depicted above the bar graph, with *stcA* shown in red to match the corresponding bar color in the graph.

**(B)** Transcript levels are presented as fold-change relative to the control (*A. nidulans* solo-culture), normalized to the constitutively expressed *actin* gene (*actA*) of *A. nidulans*. Data represent means from three independent biological replicates, each with three technical replicates. Error bars indicate standard deviation (+ SD). The statistical significance compared to the control is indicated by asterisk (* *p* < 0.05, ** *p* < 0.01, *** *p* < 0.001).


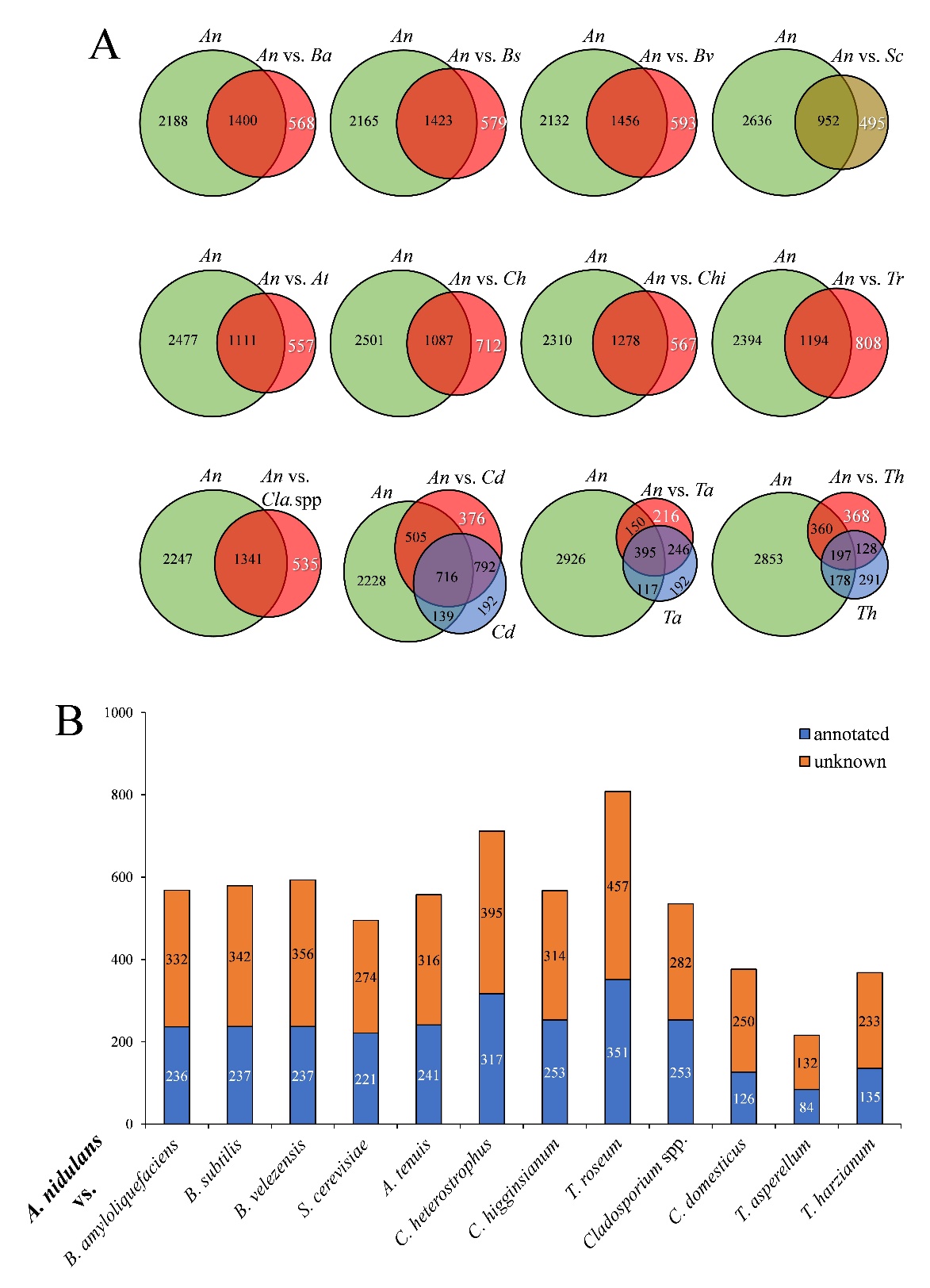


**Supplemental Figure S7. Metabolomic profiling of *A. nidulans* reveals confrontation-specific SM production.**

**(A)** Venn diagrams illustrating the number of metabolite features detected in *A. nidulans* grown alone (green circles) versus in confrontation with various bacterial and fungal species (red or olive green circles). The overlapping regions indicate shared metabolite features, while non-overlapping regions represent metabolites uniquely induced during confrontation. In confrontations with *Coprinellus domesticus* (*Cd*), *Trichoderma asperellum* (*Ta*), and *T. harzianum* (*Th*), where fungal overgrowth was observed, solo metabolomes of the confrontation partners were included (blue circles) to distinguish *A. nidulans*-specific metabolites. *An*, *A. nidulans*; *Ba*, *B. amyloliquefaciens*; *Bs*, *B. subtilis*; *Bv*, *B. velezensis*; *Sc*, *S. cerevisiae*; *At*, *A. tenuis*; *Ch*, *C. heterostrophus*; *Chi*, *C. higginsianum*; *Tr*, *T. roseum*; *Cla* spp., *Cladosporium* spp.; *Cd*, *C. domesticus*; *Ta*, *T. asperellum*; *Th*, *T. harzianum*.

**(B)** Stacked bar plots showing the number of metabolite features identified as either annotated (blue) or unknown (orange) for each confrontation. A large proportion of induced metabolites remains uncharacterized, highlighting the potential for novel SM discovery.
